# Association between poor mental health in mothers and child stunting: a population-based cross-sectional study in Rwanda

**DOI:** 10.1136/bmjopen-2025-101117

**Published:** 2025-10-13

**Authors:** Jean Nepo Utumatwishima, Ingrid Mogren, Kristina Elfving, Aline Umubyeyi, Gunilla Krantz

**Affiliations:** 1 Department of Public Health and Community Medicine, University of Gothenburg, Gothenburg, Sweden; 2 University of Rwanda, Kigali, Rwanda; 3 Department of Clinical Sciences, Obstetrics and Gynecology, Umeå University, Umeå, Sweden; 4 Department of Pediatrics, University of Gothenburg, Gothenburg, Sweden

**Keywords:** MENTAL HEALTH, Child, NUTRITION & DIETETICS, PUBLIC HEALTH, EPIDEMIOLOGY

## Abstract

**Abstract:**

**Objective:**

Child undernutrition is linked to substantial national economic and health losses in low- and middle-income countries, including Rwanda. Although the causal and contextual factors contributing to chronic malnutrition in children in Rwanda have been explored, the role of the mothers’ mental health has not been fully investigated. This study aims to determine the prevalence of major depressive disorders, generalised anxiety and suicide risk among mothers in Rwanda and to explore their association with child stunting.

**Design:**

This study used a cross-sectional, population-based design.

**Setting and participants:**

Participants included children aged 1–36 months (n=601) and their mothers (n=601) in Rwanda’s Northern Province. Mothers’ mental health was assessed using four modules from the Mini International Neuropsychiatric Interview, based on the Diagnostic and Statistical Manual of Mental Disorders. Child anthropometric measurements followed WHO guidelines.

**Primary and secondary outcomes:**

The primary outcome of the study was child stunting that was defined as a height-for-age Z (HAZ) score <−2 SD according to WHO growth standards.

**Results:**

Among the 601 mothers assessed, generalised anxiety disorder had the highest prevalence (36.6%), followed by recurrent major depressive disorder (27.3%), current major depressive disorder (22.7%) and current suicide risk (18.2%). Among the children, 27.1% were stunted, with prevalence rising from 9.8% in infants (1–12 months) to 39.9% in toddlers (25–36 months). Current major depressive disorders in mothers were associated with child stunting (adjusted OR 1.67; 95% CI 1.06 to 2.61). Affected children had lower HAZ scores (−1.68 ± 1.36 vs −1.30 ± 1.09; p = 0.004), and excess relative risk (ERR) analysis confirmed depression as a significant risk factor (ERR: 1.56; p = 0.005).

**Conclusions:**

Mental health disorders in mothers, especially depression, showed a significant association with child stunting. Addressing mental health disorders in mothers is essential for improving child nutritional outcomes.

**STRENGTHS AND LIMITATIONS OF THIS STUDY**

- It is one of the first population-based studies to provide a comprehensive assessment of mothers’ mental health, specifically examining depression, anxiety and suicide risk in relation to child stunting among children in Rwanda.
- This study also focuses on a vulnerable population in a resource-limited setting, where mothers’ mental health and child growth issues are often under-researched.
- Internationally recognised data collection tools, previously validated in similar settings, were used in this study.
- Given the sensitive nature of mental health reporting, under-reporting of certain feelings remains a possibility.
- This study’s cross-sectional design limits the ability to infer causality, and the reliance on self-reported data for mothers’ mental health may introduce reporting bias.

## Introduction

 Child stunting, also known as chronic undernutrition, is defined as a height-for-age Z (HAZ) score below −2 SD from the median of the WHO child growth standards and affects 22% of all children in the world.^1^ Stunting is measured by the HAZ score.^2^ Several studies in sub-Saharan Africa identify diverse risk factors for child stunting. Mothers’ characteristics such as low education, low weight and height and inadequate antenatal care attendance are risk factors that increase the likelihood of child stunting. Similarly, poor sanitation, large family size, higher birth order, low birth weight, suboptimal breastfeeding practices, unsafe cooking environments and low socioeconomic status contribute to a higher risk of stunting.^3^,^5^

Mental health in mothers refers to the emotional, psychological and social well-being of a woman during the preconception period, throughout pregnancy and in the postpartum period.^6^ It encompasses a mother’s ability to cope with the challenges of motherhood, form positive relationships with her child, function effectively in her daily life and feel a sense of well-being.^6^

Analysis of a large, population-representative dataset from the German Socio-Economic Panel reveals an association between poor maternal mental health and an increased risk of preterm birth and low birth weight in the offspring.^7^

A study across four countries (Peru, Ethiopia, India and Vietnam) examined the link between maternal mental health and child nutrition. The findings show that mothers with poorer mental health had children who were more likely to be stunted or underweight.^8^ A study from Rio de Janeiro finds that mothers with more severe mental health issues, such as depression, had infants with significantly lower weight and growth measurements at the age of 6 months, highlighting the link between maternal mental well-being and infant nutrition.^9^ A study from Rwanda examining mental health in mothers of children with perinatal complications, such as preterm birth, low birth weight or hypoxic ischaemic encephalopathy, reveals a high prevalence of poor mental health, affecting 50% of the mothers.^10^

Public health researchers suggest that poor mental health of mothers can have a negative impact on child growth through several mechanisms, including reduced caregiving capacity, poor decision-making and limited access to healthcare.^11^ For example, depression or anxiety can make it challenging for mothers to fulfil their child’s basic needs, such as preparing nutritious meals or ensuring proper feeding practices.^12^ Furthermore, mental health struggles may cloud a mother’s judgement when it comes to food budgeting and prioritising her child’s nutritional needs.^13^ This lack of prioritisation can also extend to healthcare utilisation. Mothers with poor mental health may be less likely to seek preventive care for their children, address potential nutritional deficiencies or identify irregularities in breastfeeding practices.^14^

Several factors contribute to the vulnerability of women’s mental health in Rwanda. These include high poverty rates, low educational attainment, exposure to negative life events and marital difficulties.^10^ The presence of depression and anxiety during pregnancy can be particularly detrimental. A study conducted in Rwanda’s Eastern Province in 2020 revealed high prevalence rates: 37.6% for depression and 28.2% for anxiety.^15^ Rwanda faces a critical challenge to address women’s mental health and child stunting.

Despite various interventions, recent research from Rwanda shows that the prevalence of stunting remains high. A recent study reveals that 34.1% of children aged 6–23 months from poor households were stunted.^16^ This is particularly concerning because the goal in Rwanda is to reduce the rate of stunting to 19% by 2024.^17^

Similar to other countries, several factors contribute to stunting in Rwanda, including the family’s socioeconomic status, household characteristics, maternal health and environmental conditions. For example, children from households without vegetable gardens, those aged 13–23 months and those with mothers who have experienced intimate partner violence (IPV) are at a higher risk of stunting.^16^ Specifically, poverty and limited decision-making power among mothers in the Northern Province of Rwanda contribute significantly to child stunting, as demonstrated in our previous study using the same database as this study.^18^ Although previous research has explored the risk factors that contribute to child stunting, the role of maternal mental health in this complex issue remains understudied within the Rwandan context. To date, no studies in Rwanda have specifically assessed the association between mothers’ mental health and child stunting using population-based data. Additionally, current national guidelines for addressing child stunting in Rwanda do not include mothers’ mental health screening or intervention as a component of prevention or treatment. This represents a significant gap, particularly given the high burden of both mothers’ mental health disorders and child stunting in the country. This study aims to contribute to a more comprehensive understanding of child stunting by focusing on the association between mental health in mothers and child stunting. By generating context-specific evidence, this research can inform the integration of mothers’ mental health services into Rwanda’s child nutrition and stunting prevention strategies and support evidence-based policymaking towards achieving national health and development goals.

## Methods

### Study design, study population and sample size

A cross-sectional study was undertaken to examine a sample of mother-child pairs in the Northern Province of Rwanda. Participants included children aged 1–36 months (n=601) and their mothers (n=601). This age range was chosen to capture both early and ongoing risk of stunting during a biologically vulnerable period. While the first 1000 days (up to 24 months) are critical for growth, evidence shows that growth faltering can continue beyond 24 months, especially in low-resource settings. Children under 1 month were excluded to avoid confounding from birth-related factors such as prematurity or low birth weight, and children over 36 months were excluded to reduce bias from external caregiving influences and to ensure closer alignment between mothers’ current mental health and child growth outcomes.

Sample size was determined using the estimated prevalence of child stunting in Rwanda, aiming for a 4% margin of error with a 95% CI.

A total of 630 households were randomly selected across the province’s five districts. A multistage sampling approach was used to identify eligible households. Initially, a random selection of 186 villages from the province’s 2743 villages was conducted using a geospatial grid. Subsequently, households within these selected villages were sampled proportionally according to size. In each chosen household, a mother with a child aged between 1 and 36 months was invited to participate in the study.

Community health workers enumerated all households with eligible mothers within their respective villages, creating a sampling frame for each village. The initial participant in each village was determined through random selection. Subsequent households were identified using a systematic sampling approach, with the sampling interval calculated based on the desired sample size for each village. In cases where the target household was inaccessible, the nearest eligible household fulfilling the inclusion criteria was included under the assumption of similar living conditions. The overall sampling strategy aimed for random village distribution and optimal spatial coverage for subsequent geographic information system (GIS) and spatial analyses.

### Data collection procedures

A comprehensive data collection instrument was designed to capture a wide range of variables associated with undernutrition, including sociodemographic, psychosocial, mother and child health, food access, anthropometrics, GIS and livestock-related factors (online supplemental file 1—questionnaire). The questionnaire was translated into Kinyarwanda and underwent a three-step validation process. First, the questionnaire was translated by a professional translator to ensure accuracy and clarity. Second, the survey was administered to a group of interviewers who were native Kinyarwanda speakers to assess the comprehensibility and cultural relevance of the instrument. Third, before the main data collection, experienced interviewers conducted a pre-test of the questionnaire with mothers and their children in a village located in the Northern Province of Rwanda to further evaluate its applicability and effectiveness in the field.

The instrument was integrated into an Android-based application using the emGeo platform to enhance data management and efficiency. The University of Rwanda’s School of Public Health led the implementation of the survey. A team of 13 experienced data collectors, comprising healthcare professionals and research trainees, conducted the fieldwork under the supervision of Rwandan and Swedish supervisors. A rigorous training programme, including a 5-day pre-training phase and a 2-day pilot in a Northern Province village, preceded data collection from November to December 2021.

### Measurement of dependent variables

Children’s height measurements were used to derive Z scores by comparing each child’s anthropometric measurements with the WHO child growth standards for age and gender.^19^ HAZ scores were calculated to assess child growth status. Stunting was defined as HAZ <−2.^19^ Anthropometric measurements, including height, were collected using standardised protocols. For children <2 years, height was measured in a recumbent position using a ShorrBoard; standing height was measured for older children. In this study, stunting, defined as a condition where a child’s HAZ was more than 2 SD below the median of the reference population, indicating chronic undernutrition, serves as the primary outcome parameter.

### Measurement of independent variables

Sociodemographic factors were categorised and analysed as independent risk factors. The age of the mother was captured as a continuous variable and later categorised into three categories (≤20, 21–34 and ≥35 years of age). Marital status included five categories: cohabitation, divorced or separated, married, single mother and widow. Marital status was dichotomised into married (or cohabiting) and living alone (being single, divorced, widowed or separated). Data about educational level were collected as never attended school, incomplete primary school, complete primary school and secondary school or more. It was then categorised into three groups: never attended school, primary level and secondary level or above. Occupation was categorised into skilled workers, students and non-skilled workers. Household income per month was categorised into two groups: households with financial income ≤36 000 Rwandan Francs (RWF) and those with >36 000 RWF. Age of the child was categorised into three categories: 1–12 months, 13–24 months and 25–36 months. The child’s birth weight, recorded in kg on the vaccination card, was categorised as low birth weight (<2.5 kg) and normal birth weight (≥2.5 kg). Child breastfeeding status was assessed with the question: ‘Is your child still breastfeeding currently?’ Responses were recorded as ‘Yes’ or ‘No’.

Building on previous findings that linked IPV to child stunting using the same database as this study, we adjusted for IPV to examine its association with maternal mental health disorders and its influence on the relationship between maternal mental health and child growth outcomes.^20^

### Assessment of mental health disorders in mothers

The mental health status of mothers was assessed using four modules of the Mini International Neuropsychiatric Interview (MINI) Diagnostic and Statistical Manual of Mental Disorders (DSM-IV) V.5.0.0. These modules included current and past major depression episodes, generalised anxiety disorder and suicide risk. The MINI, a standardised diagnostic tool aligned with DSM-IV and International Classification of Diseases, 10th revision criteria, has demonstrated comparable validity and reliability with the WHO Composite International Diagnostic Interview in previous research.^21^ The MINI’s brevity and streamlined administration make it suitable for use by trained interviewers, without requiring specialised clinical expertise. This instrument has been used successfully in previous studies conducted in Rwanda.^22^

#### Current major depression disorder (MDD)

From MINI, MDD was assessed using a nine-item screening tool consisting of three main questions (A1, A2 and A3) and seven sub-questions within question A3. Each question required a ‘Yes’ or ‘No’ response. Participants were diagnosed with MDD if they responded affirmatively to five or more of these nine items. For participants diagnosed with current MDD, a follow-up inquiry was conducted to determine if they had experienced previous depression episodes.

#### Past MDD

Past MDD was diagnosed if participants reported a history of depression symptoms lasting at least 2 weeks or if there was <2 months between the current and previous depression episodes.

#### Current generalised anxiety

To assess generalised anxiety, participants were asked a series of six questions about experiencing anxiety most of the time during the past 6 months. Responses were ‘Yes’ or ‘No’. A diagnosis of generalised anxiety was made if participants responded affirmatively to three or more of the anxiety-related questions.

#### Current suicide risk

Current suicide risk was assessed using a nine-item scale. A positive response to any of these items indicated a current suicide risk.

### Covariates

**Figure 1:**
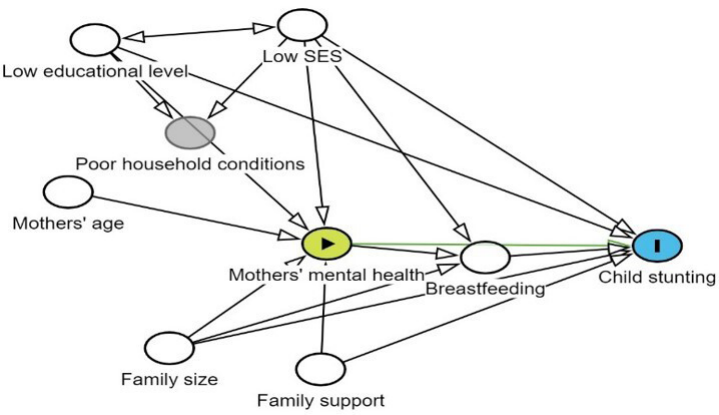
Directed acyclic graph (DAG) illustrating hypothesised pathways linking mothers’ mental health and child stunting. SES, socioeconomic status.

Using a directed acyclic graph (figure 1), socioeconomic status may influence both maternal mental health and child stunting.^23 24^ This is a confounding pathway because socioeconomic status can affect the mother’s mental health (due to stress, financial difficulties) and simultaneously affect child nutrition (due to food insecurity and access to healthcare).^23^ Household factors, such as living conditions or poor hygiene, may have an impact on maternal mental health and child nutrition.^18 23 25 26^ A mother’s educational level may affect her mental health (through empowerment or access to resources) and child stunting (by having an impact on caregiving practices and knowledge of child nutrition).^27^,^30^ The mother’s age might influence her mental health (younger mothers may experience more stress or depression) and be related to child stunting (due to inexperience or lack of resources).^31^,^33^ Adequate family support can mitigate mental health problems and provide additional resources or care for the child, potentially reducing the risk of stunting.^18^ Family size can affect maternal mental health and child stunting; larger families in poor households often have increasing caregiving demands, financial stress and resource constraints.^34^ Furthermore, children in larger families are more likely to experience stunting because scarce resources, such as food and healthcare, may be inadequate to meet their nutritional and developmental needs.^35^ A mother’s mental health, such as depression or anxiety, can reduce the duration and quality of breastfeeding, thereby limiting a child’s growth potential.^36 37^

### Statistical analysis

Prevalence (number and percentage) was used to describe the participants’ characteristics. A Venn diagram was created to illustrate the overlap and distribution of participants experiencing depression, anxiety and suicide risk. Each circle represents one of these conditions, and the overlapping areas show participants who meet the criteria for multiple conditions. The diagram displays both the number and percentage of participants in each category.

The dependent variable was child stunting, dichotomised into stunted and not stunted. HAZ scores were used to create a binary stunting variable. Children with HAZ values below −2 SD were classified as stunted (stunting=1), while those with HAZ ≥−2 were classified as not stunted (stunting=0). Missing HAZ values were handled using listwise deletion, meaning that any observation with missing data on HAZ or relevant variables was excluded from analyses involving the stunting variable.

The distribution of sociodemographic variables in relation to mental health disorders in Rwandan mothers and the test of difference for two groups were calculated by applying Pearson’s χ^2^ test. Associations between mothers’ mental health disorders and child stunting were estimated by multivariable logistic regression models. Interactions between mothers’ mental health disorders and child stunting were assessed using the excess relative risk (ERR), relative excess risk due to interaction (RERI=RR_11_−RR_10_−RR_01_+RR_00_), attributable proportion (AP=RERI/RR_11_) and the synergy index (SI=RR_11_−1/(RR_10_−1)+(RR_01_−1).^38^ RR_11_, RR_10_ and RR_01_ represent relative risks for specific exposure combinations. RR_11_ is the relative risk when both exposures (X_1_ and X_2_) are present, RR_10_ is the relative risk when only X_1_ is present and RR_01_ is the relative risk when only X_2_ is present. These are compared with RR_00_, the baseline relative risk with no exposures (X_1_=0 and X_2_=0).^38^

ERR quantifies the combined effect of exposures beyond their independent effects, and RERI measures additive interaction. AP represents the proportion of the outcome attributable to the interaction, and the synergy index evaluates multiplicative interaction.

Statistical significance was determined using Z scores and p values; SEs were calculated for ERR and RERI.

P values ≤0.05 were considered statistically significant and measures of association are presented as ORs with their 95% CI. All analyses were performed using STATA V.17 SE.

### Patient and public involvement statement

Patients and/or members of the public were not directly involved in the design or conduct of this study. However, the study was informed by national priorities and public health needs identified through previous stakeholders’ consultations, and its findings will be disseminated to relevant community groups and health policymakers.

## Results

### Sociodemographic characteristics and prevalence of mental health disorders

The participation rate was 95.4% (n=601). There were incomplete data for 29 mother-child pairs. Of the 601 mothers who participated in the study, the prevalence of the four mental health disorders analysed was as follows: current generalised anxiety disorder (36.6%), recurrent MDD (27.3%), current MDD (22.7%) and current suicide risk (18.2%). Mothers aged ≤20 years were significantly more likely to experience generalised anxiety disorder (p=0.024), although no other statistically significant associations were found with age for other mental health conditions. Most of the mothers reported a primary-level education (68.5%), and 9.7% reported never attending school, but educational attainment showed no statistically significant association with any of the mental health disorders (online supplemental table 1). Most of the mothers in the study were married (88.3%). A statistically significant association was observed between household income and mental health; mothers from households earning <36 000 RWF were more likely to experience current MDD (p=0.014), recurrent MDD (p=0.002), current suicide risk (p=0.022) and generalised anxiety disorder (p=0.003). In addition, mothers whose children had a birth weight <2.5 kg demonstrated higher rates of mental health issues across all conditions (online supplemental table 1).

### Anthropometric status of the children

Among the 601 children, 51.7% were boys (n=311) and 48.3% were girls (n=290). Approximately one-third of the children were aged 1–6 months, another one-third were aged 13–24 months and the remaining one-third were aged 25–36 months. The birth weight of 89.7% of the children was <2.5 kg (online supplemental table 1). Child stunting increased with age based on the measurements (table 1).

**Table 1:** Anthropometric indicators and prevalence of malnutrition by age group (n=601)

	Child’s age		
	1–12 months	13–24 months	25–36 months
Z score, mean±SD			
Height-for-age	−0.81±1.21	−1.64±1.25	−1.77±1.13
Weight-for-age	−0.34±1.12	−0.54±0.99	−0.60±0.97
Weight-for-height	0.27±1.27	0.34±1.12	0.51±1.06
Prevalence, % (95% CI)			
Stunting	9.8 (7.6 to 12.5)	34.3 (30.5 to 38.2)	39.9 (35.9 to 43.9)
Underweight	6.1 (4.4 to 8.4)	7.0 (5.0 to 9.3)	7.5 (5.5 to 9.9)
Wasting	3.7 (2.3 to 5.5)	3.3 (2.0 to 5.1)	1.2 (0.5 to 2.4)

CI, confidence interval; SD, standard deviation.

### Co-occurrence and overlap among depression, anxiety and suicide risk

**Figure 2:**
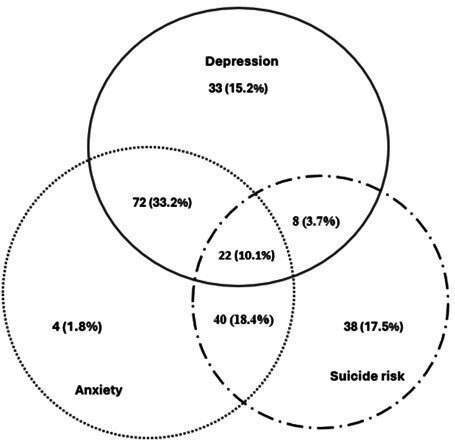
Venn diagram illustrating the overlap of depression, anxiety and suicide risk among mothers, with the number and percentage of participants experiencing one or more of these conditions.

Of the 601 mothers, 217 (36.1%) reported experiencing at least one of the mental health disorders investigated (figure 2). A notable overlap was observed between depression and anxiety; 72 individuals (33.2%) were diagnosed with both conditions. Furthermore, suicide risk was increased among individuals with both depression (30 individuals; 13.8%) and anxiety (62 individuals; 28.5%). A substantial number of participants exhibited one or more of these conditions without the others, highlighting the diversity of mental health disorders within this population.

### Mental health disorders in mothers and their association with child stunting

Among the 601 children included in the study, 27.1% (n=163) were stunted. The crude odds of having a stunted child among mothers with current MDD were 1.71 (95% CI 1.14 to 2.59). After adjusting for the mother’s age, education, household income and social support, the adjusted OR was statistically significant for MDD only (table 2).

**Table 2:** Association between mothers’ mental health disorders and child stunting (n=601)

Mental health disorders	Mothers exposed, n (%)	Child stunting, n (%)		Crude OR	Model 1*	Model 2†
		Yes (n=163)	No (n=438)			
Major depression disorder current						
No	460 (77.3)	112 (70.0)	348 (80.0)	1		
Yes	135 (22.7)	48 (30.0)	87 (20.0)	**1.71 (1.14–2.59)**	**1.67 (1.06–2.61)**	
Physical IPV	–	–	–	–		**1.81 (1.17–2.81)**
Sexual IPV	–	–	–	–		**1.67 (1.08–2.60)**
Mental IPV	–	–	–	–		**1.61 (1.04–2.48)**
Major depression episode recurrent						
No	216 (72.7)	61 (67.8)	155 (74.9)	1		
Yes	81 (27.3)	29 (32.2)	52 (25.1)	**1.42 (1.01–2.44)**	1.39 (0.81–2.42)	
Physical IPV	–	–	–	–		1.41 (0.78–2.53)
Sexual IPV	–	–	–	–		1.30 (0.72–2.33)
Mental IPV	–	–	–	–		1.22 (0.68–2.18)
Current suicide risk						
No	485 (81.8)	133 (83.7)	352 (81.1)	1		
Yes	108 (18.2)	26 (16.3)	82 (18.9)	0.84 (0.52–1.36)	0.85 (0.52–1.39)	
Physical IPV	–	–	–	–		0.84 (0.50–1.39)
Sexual IPV	–	–	–	–		0.76 (0.45–1.27)
Mental IPV	–	–	–	–		0.75 (0.45–1.26)
Generalised anxiety disorder current						
No	239 (63.4)	70 (61.9)	169 (64.0)	1		
Yes	138 (36.6)	43 (38.1)	95 (36.0)	1.09 (0.69–1.72)	1.05 (0.66–1.67)	
Physical IPV	–	–	–	–		1.07 (0.66–1.72)
Sexual IPV	–	–	–	–		1.01 (0.62–1.63)
Mental IPV	–	–	–	–		0.96 (0.59–1.55)

Values in bold type are statistically significant.

* Model 1: crude OR adjusted for mother’s age, education, household income, breastfeeding duration and social support.

† Model 2: crude OR adjusted for intimate partner violence (IPV).

For mothers with a history of MDD, the crude odds of having a stunted child were 1.42 (95% CI 1.01 to 2.44), but this association did not remain statistically significant after adjustment. Mothers exposed to current suicide risk and those with generalised anxiety disorder had non-significant ORs. Therefore, only MDD in the mother was statistically significantly associated with stunting in the offspring in this population (table 2).

### Risk of child stunting due to interactions between different mental health disorders

The findings presented in table 3 highlight the excess risk of child stunting due to interactions between different mental health disorders in the mothers. The interaction between depression and suicide showed a statistically significant positive ERR for stunting when depression was present, but suicide risk was absent (ERR=1.38, p=0.002). However, the interaction between depression and anxiety did not show a statistically significant excess risk (ERR=0.46, p=0.158). The interaction between suicide and anxiety did not reveal any statistically significant associations. When adjusting for anxiety, the combination of depression and the absence of suicide risk continued to show a statistically significant excess risk (ERR=1.56, p=0.005), further reinforcing the detrimental impact of maternal depression on child stunting (table 3).

**Table 3:** Excess risk of child stunting due to interactions between different mental health disorders in mothers

Interactions of mental health disorders	ERR	SE	Z score	P value	RERI	Attributable proportion	Synergy index
Depression (+)/suicide (−)					−0.87	−0.78	0.13
−+	−0.38	0.29	−1.03	0.302			
+−	1.38	0.67	3.09	0.002			
++	0.13	0.32	0.32	0.664			
Depression (+)/anxiety (−)					−0.14	−0.09	0.77
−+	−0.32	0.27	−0.95	0.343			
+−	0.91	0.76	1.64	0.101			
++	0.46	0.39	1.41	0.158			
Suicide (+)/anxiety (−)					−0.13	−0.14	–
−+	0.19	0.34	0.60	0.549			
+−	0.16	0.42	−0.35	0.723			
++	0.10	0.29	−0.34	0.736			
Depression (+)/suicide (−) adjusted for anxiety					−0.77	−0.57	0.32
−+	0.44	0.37	−0.88	0.380			
+−	1.56	0.86	2.78	0.005			
++	0.36	0.49	0.84	0.401			

ERR, excess relative risk; RERI, relative excess risk due to interaction.

### Association between depression symptoms in mothers and nutritional status of children

Of the 601 mothers included in the study, 60% (n=363) responded to questions assessing depression symptoms. Of those 363, 40.2% (n=146) confirmed having depression symptoms. The anthropometric measurements (height-for-age) of the children of mothers with depression symptoms revealed a mean Z score of −1.68±1.36, whereas mothers without depression symptoms showed a lower mean Z score of −1.30±1.09. The difference was statistically significant (p=0.004). Similarly, children of mothers with depression were underweight, and the difference in mean Z scores was statistically significant (p<0.001).

### Mental health symptoms in mothers and their association with child stunting

Of the mothers interviewed, 40.2% (n=146) had at least one depression symptom. The distribution of stunting status among the children of these mothers was higher (51.9%; n=54) compared with children of mothers without depression symptoms (48.1%; n=50); the crude OR was 1.96 (95% CI 1.24 to 3.11). Regarding suicide ideation, 18.2% (n=108) of mothers reported at least one symptom. At least one anxiety symptom was reported by 52.6% (n=179) of mothers. No statistical significance was found between mothers with at least one symptom of suicide ideation or anxiety and child stunting. Mothers who experienced a reduced appetite and sleep disturbances had higher odds of having stunted children (OR 1.64; 95% CI 1.01 to 2.66 vs OR 1.59; 95% CI 1.03 to 2.58, respectively) (table 4).

**Table 4:** Association between depression symptoms in mothers and child stunting

Variables	Mothers exposed, n (%)	Child stunting, n (%)		Crude OR
		Yes (n=163)	No (n=438)	
Reduced or changed appetite/weight				
No	204 (62.8)	54 (54.6)	150 (66.4)	1
Yes	121 (37.2)	45 (45.5)	76 (33.6)	**1.64 (1.01–2.66)** *
Sleep disturbances				
No	204 (63.2)	55 (55.6)	149 (66.5)	1
Yes	119 (36.8)	44 (44.4)	75 (33.5)	**1.59 (1.03–2.58)**
Psychomotor changes (talk or move slowly)				
No	246 (75.5)	70 (71.4)	176 (77.2)	1
Yes	80 (24.5)	28 (28.6)	52 (22.8)	1.35 (0.79–2.31)
Fatigue/energy loss				
No	178 (62.0)	55 (59.1)	123 (63.4)	1
Yes	109 (38.0)	38 (40.9)	71 (36.6)	1.19 (0.72–1.99)
Feelings of worthlessness/guilt				
No	195 (67.0)	65 (68.4)	130 (66.3)	
Yes	96 (33.0)	30 (31.6)	66 (33.7)	0.91 (0.54–1.53)
Difficulty concentrating/deciding				
No	196 (66.8)	61 (64.9)	132 (67.7)	1
Yes	96 (33.2)	33 (35.1)	63 (32.3)	1.13 (0.67–1.90)
Suicidal thoughts/ideation				
No	238 (82.1)	77 (81.1)	161 (82.6)	1
Yes	52 (17.9)	18 (18.9)	34 (17.4)	1.11 (0.59–2.08)
Previous depression episodes				
No	216 (72.7)	61 (67.8)	155 (74.9)	1
Yes	81 (27.3)	29 (32.2)	52 (25.1)	1.42 (0.82–2.44)

* Values in bold type are statistically significant.

## Discussion

This study underscores that mental health disorders in mothers are a critical public health concern associated with child undernutrition in Rwanda. The main finding of this study was the association between mothers’ depression and child stunting, emphasising the critical role of mothers’ mental health in optimising child growth and development.

A substantial proportion of mothers who participated in the study reported mental health challenges, including generalised anxiety, recurrent and current MDDs and suicide risk. Notably, younger, poor and unmarried mothers experienced a disproportionate impact.

The literature consistently identifies current MDD as the most prevalent mental health disorder in mothers globally, with considerable variation in prevalence rates across low- and middle-income countries.^8 39^ The high prevalence of mental health disorders in mothers in low-income countries might be due to lower socioeconomic conditions, food insecurity, low educational level, unsupportive members of households including partners and experiencing physical violence.^38^ The association between mothers’ depression and poor nutritional status in Rwanda is confirmed by the evidence from other countries.^40 41^

Children born to mothers experiencing depression demonstrated significantly lower HAZ scores compared with those born to non-depressed mothers. Stunting reflects chronic undernutrition, indicating long-term deprivation of essential nutrients.^42^ This finding contributes to a growing body of international evidence linking mothers’ mental health and child linear growth. For instance, a multi-country study conducted in Peru, Ethiopia, India and Vietnam found that children of mothers with poor mental health were more likely to be stunted.^8^ Similarly, research from Brazil (Rio de Janeiro) highlighted that maternal depression was associated with lower growth measurements in infants.^9^ In Rwanda, studies among mothers of children with perinatal complications also revealed a high prevalence of poor maternal mental health, further reinforcing the importance of psychological well-being in influencing child growth outcomes.^10^

The association observed between mothers’ depression and child stunting aligns with the findings of Wemakor *et al* in Ghana, suggesting that this relationship may be prevalent across similar populations.^43^ The consistent association between mothers’ depression and child stunting across low- and middle-income countries may be attributed to shared sociocultural factors influencing caregiving, feeding practices, maternal education, socioeconomic status, household food security and other related determinants.^44^

Childcare practices significantly influence child nutrition outcomes. A key factor affecting these practices is the caregiver’s mental health, as emphasised in the UNICEF care model.^45^ Child stunting may be linked to mothers’ depression through the intermediary factor of suboptimal feeding practices.^46^ Specific symptoms of depression, including reduced appetite and sleep disturbances, were independently associated with increased odds of child stunting, suggesting that interventions targeting these symptoms could be particularly effective in preventing poor child growth.^47^

Although anxiety symptoms were prevalent in the study population, they did not have a statistically significant impact on child stunting, highlighting the specificity of depression in relation to child growth outcomes.

Although suicide ideation was common among mothers, no direct association with child stunting was found. Given the severity of suicide ideation, further research is warranted to explore its potential indirect effects on child health outcomes. Depression symptoms have been found consistently in Rwandan mothers and are associated with increased IPV, lack of social support and poverty.^18 48 49^ The present study underscores the importance of investigating suicide ideation among Rwandan mothers as a specific factor influencing child stunting. To our knowledge, this is the first study to explore suicidal risk as a potential risk factor for child undernutrition. The most notable finding in our study is the significant positive association between depression and child stunting, particularly when suicide risk is absent.

This suggests that mothers’ depression alone is a critical driver of stunting, with an ERR of 1.38, indicating that children of mothers with depression, but without suicide risk, have a substantially higher likelihood of being stunted. This highlights the potentially severe impact of untreated or unmanaged depression of mothers on child growth and development. The interaction between depression and anxiety did not reach statistical significance. This suggests that although anxiety in isolation may not independently increase the risk of stunting, it may exacerbate the effect of depression. The interaction between suicide risk and anxiety showed no meaningful associations with the risk of stunting. This may suggest that anxiety, coupled with suicide risk, does not significantly alter the likelihood of stunting. It is possible that depression, rather than anxiety or suicide risk, plays a more direct and measurable role in the nutrition and development of children.

Although the age-adjusted suicide rate in Rwanda of approximately 12 deaths per 100 000 persons per year may seem relatively low, the potential for under-reporting and the disproportionate impact of mental health on women necessitate further exploration of this critical issue.^50^ It is well-established in mental health research that for every completed suicide, there are approximately 20 suicide attempts. This suggests that a significantly larger population experience suicidal ideation or planning, emphasising the importance of addressing these precursors to suicide.^50^ Moreover, the well-established link between depression and suicide underscores the complexity of this issue. Depression is a significant risk factor for suicidal behaviour, highlighting the need for comprehensive mental health interventions.^51^ The prevalence of IPV among mothers in rural Rwanda is also another critical concern. Previous research, such as the work of Umubyeyi *et al* has demonstrated a strong association between IPV and various common mental health disorders in mothers.^22^ In our previous work, we demonstrated that mothers exposed to IPV are more likely to have children with stunted growth.^20^ After adjusting for IPV, the OR for having stunted children remained statistically significant among those exposed to both current MDD and all categories of IPV.

### Methodological considerations

This study has several notable strengths. It is one of the first population-based studies to provide a comprehensive assessment of mothers’ mental health, specifically examining depression, anxiety and suicide risk in relation to chronic undernutrition among children in Rwanda. It also focuses on a vulnerable population in a resource-limited setting, where mothers’ mental health and child growth issues are often under-researched. The use of standardised tools for assessing mental health disorders and child stunting, as well as adjusting for important sociodemographic confounders, further strengthens the study’s validity. However, the study’s cross-sectional design limits the ability to infer causality, and the reliance on self-reported data for mothers’ mental health may introduce reporting bias. Unmeasured confounders, such as maternal nutrition and healthcare access, were not accounted for, and the study’s focus on mothers’ mental health alone overlooks the potential influence of paternal mental health or broader family dynamics. The exclusion of mothers with missing anthropometric or mental health data through listwise deletion may have led to selection bias, potentially affecting the generalisability of the findings. The study was also limited to rural settings, which may not reflect the situation in urban areas, where risk factors and health service access could differ.

## Conclusions

This study underscores the association between depression in mothers and child stunting in Rwanda, showing that children of depressed mothers had lower HAZ scores, reflecting chronic undernutrition. Depression emerged as a key factor affecting child growth, while anxiety and suicide risk did not. To address this, integrating mental health services into antenatal and postpartum care, alongside nutritional programmes, may help support both mothers and their children. Training in feeding practices, building community support and raising awareness about maternal mental health are also important steps towards improving child development.

## Supplementary material

10.1136/bmjopen-2025-101117 online supplemental file 1

10.1136/bmjopen-2025-101117 online supplemental file 2

10.1136/bmjopen-2025-101117 online supplemental table 1

## Data Availability

Data are available upon reasonable request.
