# Supplementary material for: Association between poor mental health in mothers and child stunting: a population-based cross-sectional study in Rwanda
Source: BMJ Open. 2025 Oct 13;15(10):e101117. doi: 10.1136/bmjopen-2025-101117 (PMC12519718; doi:10.1136/bmjopen-2025-101117)
Supplement: online supplemental file 1 [file bmjopen-15-10-s001.docx]

**PHD Questionnaire**

Student: Jean Nepo Utumatwishima

Gothenburg University

| a0_name_of_interviewer | Name of interviewer |  |
| --- | --- | --- |
| a0_date_of_interview | Date of interview |  |
| a0_phone_address | Phone number and/or contact address of respondent: |  |
| a0_guardian_signed_witnessed_consent | Has the respondent signed a witnessed consent? |  |
| a0_respondent | Is the respondent the mother of the household? |  |
| a0_ubedehe_category | Ubedehe category? |  |
| a1_age | A 1. What is your age (in years) (addressed to the mother)? |  |
| a1_age_dont_know | do not know | false |
| a2_deliver_alive_child_in_time_period | A 2a. Is there a child in this household with an age between 1 month up to 3 years that is still alive? |  |
| a2_twin_or_triple | A 2b. If yes in A 2a, is the child a twin or a triplet? |  |
| a3_1_respondent_names | Respondent names |  |
| a3_2_farm_id | Farm/household ID |  |
| a3_4_district | District |  |
| a3_5_cell | Cell |  |
| a3_6_sector | Sector |  |
| a3_7_village | Village |  |
| a3_8_latitude | Latitude |  |
| a3_9_longitude | Longitude |  |
| a4_maritial_status | A 4. What is your marital status? |  |
| a5_married_at_age | A 5. At what age did you get married? |  |
| a6_number_people_in_household | A 6. How many people do most of the time live in your household? |  |
| a7_number_children_birth_to | A 7. How many children did you give birth to? |  |
| a8_number_alive_children | A 8. How many are alive today? |  |
| a9_children_ages | A 9.How old are they (in years)? |  |
| a10_sex_of_household_head | A 10. What is the sex of your head of household? |  |
| a11_relationship_with_household_head | A 11. What is your relationship with the household head? |  |
| a12_religion | A 12. What is your religion? |  |
| a13_attended_school | A 13. Have you ever attended school? |  |
| a14_education_level | A 14. What level of education did you reach? |  |
| a15_total_years_school_and_university | A 15. Total number of years at school and university level |  |
| a16_main_daily_activity | A 16. What is your main daily activity? |  |
| a17_paidjob_type | A 17. If you have a paid job, are you in… ? |  |
| a18_occupation | A 18. What is your occupation? |  |
| a19_health_insurance | A 19. Do you have a health insurance? (Report the main one) |  |
| a20_partner_age | A 20. Age of your husband/partner (in years) |  |
| a21_partner_attended_school | A 21. Has your husband/partner ever attended school? |  |
| a22_partner_education_level | A 22. What level of education did your husband/partner reach? |  |
| a23_paertner_total_years_school_university | A 23. Total number of years at school and university level |  |
| a24_partner_has_job | A 24. Does your husband/partner have a paid job? |  |
| a25_partner_job_type | A 25. If he has a paid job, is he in? |  |
| a26_partner_occupation | A 26. What is your husband/partner's occupation? |  |
| a27_total_household_income | A 27. What is the total household income per month? (This should be the sum of what all household members bring in taken together) |  |
| a29_1_food | 1. Food |  |
| a29_2_health_care | 2. Health care |  |
| a29_3_rent | 3. Rent |  |
| a29_4_education | 4. Education |  |
| a29_5_transport | 5. Transport |  |
| a29_6_investment | 6. Investments |  |
| a29_7_saving | 7. Savings |  |
| a29_8_loans | 8. Loans |  |
| a30_sell_household_assets_lastmonth | A 30. In the past month, did you have to sell any household assets? If so for what reason? |  |
| a31_house_type | A 31. What type of house do you live in? |  |
| a32_drinking_water_source | A 32. What is the main source of drinking water for members of your household? |  |
| a33_how_long_take_get_water | A 33. How long does it take to get water, and come back including queueing? (Go, queue and return) |  |
| a34_treat_water_to_drink | A 34. Do you treat water to drink in any way? |  |
| a35_how_treat_drinking_water | A 35. What are you using to treat drinking water? |  |
| a36_water_used_inday_for_hygiene_drinking | A 36. How many liters of water do you use in a day for hygiene and drinking? |  |
| a38_kind_of_toilet | A 38. What kind of toilet facility does your household have? |  |
| a39_share_toilet_with_other_families | A 39. Do you share toilet facilities with other families? |  |
| a40_how_many_household_use_this_toilet | A 40. How many households use this toilet facility? |  |
| a41_have_able_wash_with_soap_last_24h | A 41. Have you been able to wash with soap and water in the last 24 hours? |  |
| a42_handwashing_place_near_toilet | A 42. Is there a place for handwashing near (within 5 m) from the toilet/latrine? |  |
| a43_what_done_to_dispose_stools | A 43. The last time (your child) passed stools, what was done to dispose of the stools? |  |
| a44_do_wash_hands_after_helping_child_defecate | A 44. Do you wash your hands after helping your child defecate? |  |
| a45_washing_hand_before_preparing_food | A 45. Do you wash your hands before preparing food? |  |
| a46_wahsing_hand_after_toilet | A 46. Do you wash your hands after using the toilet? |  |
| a47_cocking_done_inside | A 47. Is your cooking done inside the house, outside or both? |  |
| a48_access_to_electricity | A 48. Dose your household have access to electricity? |  |
| a49_bic_mot_car | A 49. Does your household have: |  |
| a50_appliances | A 50. Does any member/s of your household have: |  |
| b1_have_friend_to_assist_when_ill | B 1. Do you have a friend or family member that will assist you if you become ill? |  |
| b2_share_food_with_you | B 2. Share food with you? |  |
| b3_share_their_house_with_you | B 3. Share their house with you? |  |
| b4_lend_you_money | B 4. Lend you money |  |
| b5_help_guidance_in_problems | B 5. Help you with guidance to improve your situation when you have problems? |  |
| b6_support_in_personal_problems | B 6. Offer support to you if you run into personal problems? |  |
| b7_belong_to_any_association | B 7. Do you belong to any association, such as a cooperative, church group, women's group, youth group, sports organization? |  |
| c1_number_of_pregnancy | C 1. How many times have you been pregnant? |  |
| c2_alive_born | C 2. Number of children born alive? |  |
| c3_born_year_weight | C 3. What years were they born (live births) and what were their birth weights (in grams)? |  |
| c4_children_stillborn | C 4. Number of children that were stillborn? |  |
| c5_boys | C 5. How many of your children are boys? |  |
| c6_girls | C 6. How many of your children are girls? |  |
| c7_number_born_caesarean | C 7. How many of your children were born by caesarean section? |  |
| c8_number_born_at_health_facility | C 8. How many of your children were born at a health facility? |  |
| c9_number_miscarriages | C 9. Number of miscarriages? |  |
| c11_number_anc_visits | C 11. How many times did you go to the health centre for Antenatal care (ANC)-visits during your pregnancy with the child NAME? |  |
| c13_where | C 13. Did you go anywhere else for antenatal care ( ANC) check-ups during your pregnancy? |  |
| c15_where_give_birth | C 15. Where did you give birth? |  |
| c16_mode_of_delivery | C 16. Mode of delivery? |  |
| d0_child_name | What is child's name? |  |
| d1_birthday_day | Day |  |
| d1_birthday_month | Month |  |
| d1_birthday_year | Year |  |
| d2_child_age | D 2. How old is (NAME) (in month)? |  |
| d3_birth_certificate | D 3. Does (NAME) have a birth certificate? |  |
| d4_birth_been_registered | D 4. Has (NAME)'s birth been registered with *THE CIVIL AUTHORITIES*? |  |
| d5_know_howto_register | D 5. Do you know how to register (NAME)'s birth? |  |
| d7_child_gender | D 7. Is it a boy or girl/(Sex of child) |  |
| d8_birthweight | D 8. Birthweight (g) |  |
| d9_weight | 1. Weight of the child in grams |  |
| d9_weight of mother | 1.1_weight of the mother in Kg |  |
| d9_height | 2. Height/Length of the child |  |
| d9 _height of mother | 2.1 height or length of the mother |  |
| d9_head_circumference | 3. Head circumference |  |
| d9_mid_upper_arm_circumference | 4. Mid Upper Arm Circumference: |  |
| d9_temperature | 5. Temperature? (one decimal) |  |
| d9_respiratory_rate | 6. Respiratory rate ? (must record 2 numbers XY) |  |
| d9_haemoglobin | 7. Haemoglobin result today (one decimal) |  |
| d9_sample_collection_rectal_swab_performed | 8. Sample collection- rectal swab sample collection from the child NAME performed |  |
| d9_study_id | 9. Indicate the study-ID |  |
| d10_bipedal_edema | D 10. Does the child have swollen/edematous legs? |  |
| d10_bipedal_edema_both_legs | Are both legs swollen/edematous? |  |
| d11_visible_severe_wasting | D 11. Does the child have visible severe wasting, i.e. Is the child too thin? |  |
| d12_weight_for_height | D 12. What is the Weight-for-height (W/H) of the child? |  |
| d13_child_overal_health | D 13. Would you say that the overall health of the child today is |  |
| d15_days_left_alone_more_than_hour | D 15. Left alone for more than 15 minutes? |  |
| d16_days_left_with_another_child | D 16. Did you have to leave your child in the care of another child, that is, someone less than 10 years old, for more than an hour during the last week? |  |
| d17_read_books | D 17. Read books or looked at picture books with (name)? |  |
| d18_told_stories | D 18. Told stories to (name)? |  |
| d19_sang_songs | D 19. Sang songs to or with (name), including lullabies? |  |
| d21_played_with | D 21. Played with (name)? |  |
| d22_named_counted_draw_things_with | D 22. Named, counted, or drew things for or with (name)? |  |
| d23_can_pickup_small_object | D 23. Can (name) pick up a small object with two fingers, like a stick or a rock from the ground? |  |
| d26_have_difficulty_walking | D 26. Does (name) have difficulty walking? |  |
| d27_uses_equipment_for_walking | D 27. Does (name) use any equipment or receive assistance for walking? |  |
| d29_has_difficalty_understanding_you | D 29. Does (name) have difficulty understanding you? |  |
| d30_when_speeks_you_have_difficalty_understanding | D 30. When (name) speaks, do you have difficulty understanding (him/her)? |  |
| d32_compared_to_other_has_difficalty_playing | D 32. Compared with children of the same age, does (name) have difficulty playing? |  |
| d33_compared_to_other_how_much_hit_other | D 33. Compared with children of the same age, how much does (name) kick, bite or hit other children or adults? |  |
| d34_has_any_vaccinations | D 34. Did (NAME) ever have any vaccinations to prevent him/her from getting diseases, including vaccinations received in a national immunization day campaign? |  |
| d35_has_national_immunisation_record | D 35. Do you have a National Child Immunisation Record or immunisation records from a private health provider or any other document where (NAME)'s vaccinations are written down? |  |
| d38_is_child_ill_now | D 38. Is the child ill now? |  |
| d40_oral_intake | D 40. Oral intake? |  |
| d41_vomiting | D 41. Vomiting? |  |
| d42_ear_discharge | D 42. Ear discharge? |  |
| d43_antibiotic_last_2weeks | D 43. Antibiotic use in the last two weeks? |  |
| d45_diarrhea_in_last_2weeks | D 45. Has name suffered from diarrhea in the past two weeks |  |
| d46_blood_in_stool | D 46. Was there blood in the stool at any time? |  |
| d47_given_child_during_diarrhea | D 47. What was given to the child to drink during the time s/he had diarrhea? |  |
| d52_fever_in_last_2weeks | D 52. At any time in the last two weeks, has (name) been ill with a fever? |  |
| d53_during_illness_has_blood_testing | D 53. At any time during the illness, did (name) have blood taken from (his/her) finger or heel for testing? |  |
| d54_illness_with_cough_last_2weeks | D 54. At any time in the last two weeks, has (name) had an illness with a cough? |  |
| d55_difficulty_breathing_last2weeks | D 55. At any time in the last two weeks, has (name) had fast, short, rapid breaths or difficulty breathing? |  |
| d57_take_child_clinic_by | D 57. IF your child has an illness that you cannot manage at home on your own and you need to seek health care. What kind of transportation do you use? TRAVEL BY: |  |
| d58_hours | Hours |  |
| d58_minutes | MINUTES |  |
| d59_seek_advice_from_other_sources | D59. Did you seek any advice or treatment for the illness (diarrhea, cough or fever) from any source? |  |
| d60_1_public_medical_sector | PUBLIC MEDICAL SECTOR |  |
| d60_2_private_medical_sector | PRIVATE MEDICAL SECTOR |  |
| d60_3_dk_public_or_private | Don't know PUBLIC OR PRIVATE |  |
| d60_4_other_source | OTHER SOURCE |  |
| d60_5_other_specify | OTHER (specify) |  |
| d60_6_dk | DON'T KNOW / DON'T REMEMBER | false |
| d61_child_has_chronic_illness | D 61.Does your child have any of these diseases? |  |
| d62_child_take_medicine_everyday | D 62. Does the child take a medicine every day? |  |
| d63_which_medicine | D 63. If yes, which medicine? |  |
| e1_worried_have_enough_food | E 1. In the past four weeks, did you worry that your household would not have enough food? |  |
| e2_not_eat | E 2. In the past four weeks were you or any household member not able to eat the kinds of foods you preferred because of a lack of resources? |  |
| e3_limited_food | E 3. In the past four weeks, did you or any household member have to eat a limited variety of foods due to a lack of resources? |  |
| e4_eat_food_unwanted | E 4. In the past four weeks, did you or any household member have to eat some foods that you really did not want to eat because of a lack of resources to obtain other types of food? |  |
| e5_eat_smaller_meal | E 5. In the past four weeks, did you or any household member have to eat a smaller meal than you felt you needed because there was not enough food? |  |
| e6_fewer_meal_in_a_day | E 6. In the past four weeks, did you or any other household member have to eat fewer meals in a day because there was not enough food? |  |
| e7_no_food_to_eat | E 7. In the past four weeks, was there ever no food to eat of any kind in your household because of lack of resources to get food? |  |
| e8_goto_sleep_hungry | E 8. In the past four weeks, did you or any household member go to sleep at night hungry because there was not enough food? |  |
| e9_no_eating_day_and_night | E 9. In the past four weeks, did you or any household member go a whole day and night without eating anything because there was not enough food? |  |
| e11_borrow_food | E 11. In the past four weeks did you or any household member borrow food or rely on help from a relative or friend? |  |
| e13_mother_female_guardian_weight | E 13. Weight of the mother |  |
| e14_1_1 | Breakfast |  |
| e14_1_2 | Snack |  |
| e14_1_3 | Lunch |  |
| e14_1_4 | Snack |  |
| e14_1_5 | Dinner |  |
| e14_1_6 | Snack |  |
| e14_1_f1 | a. CEREALS | false |
| e14_1_f1_times_yesterday | How many times yesterday (past 24 hours)? |  |
| e14_1_f1_source | Main source of food |  |
| e14_1_f2 | b. WHITE ROOTS AND TUBERS | false |
| e14_1_f2_times_yesterday | How many of times yesterday (past 24 hours)? |  |
| e14_1_f2_source | Main source of food |  |
| e14_2_f1 | c. LEGUMES, NUTS AND SEEDS | false |
| e14_2_f1_times_yesterday | How many of times yesterday (past 24 hours)? |  |
| e14_2_f1_source | Main source of food |  |
| e14_3_f1 | d. VITAMIN A RICH VEGETABLES AND TUBERS | false |
| e14_3_f1_times_yesterday | How many of times yesterday (past 24 hours)? |  |
| e14_3_f1_source | Main source of food |  |
| e14_3_f2 | e. DARK GREEN LEAFY VEGETABLES | false |
| e14_3_f2_times_yesterday | How many of times yesterday (past 24 hours)? |  |
| e14_3_f2_source | Main source of food |  |
| e14_3_f3 | f. OTHER VEGETABLES | false |
| e14_3_f3_times_yesterday | How many of times yesterday (past 24 hours)? |  |
| e14_3_f3_source | Main source of food |  |
| e14_4_f1 | g. VITAMIN A RICH FRUITS | false |
| e14_4_f1_times_yesterday | How many of times yesterday (past 24 hours)? |  |
| e14_4_f1_source | Main source of food |  |
| e14_4_f2 | h. OTHER FRUITS | false |
| e14_4_f2_times_yesterday | How many of times yesterday (past 24 hours)? |  |
| e14_4_f2_source | Main source of food |  |
| e14_5_f1 | i. ORGAN MEAT | false |
| e14_5_f1_times_yesterday | How many of times yesterday (past 24 hours)? |  |
| e14_5_f1_source | Main source of food |  |
| e14_5_f2 | j. FLESH MEATS | false |
| e14_5_f2_times_yesterday | How many of times yesterday (past 24 hours)? |  |
| e14_5_f2_source | Main source of food |  |
| e14_5_f3 | k. EGGS | false |
| e14_5_f3_times_yesterday | How many of times yesterday (past 24 hours)? |  |
| e14_5_f3_source | Main source of food |  |
| e14_5_f4 | l. FISH | false |
| e14_5_f4_times_yesterday | How many of times yesterday (past 24 hours)? |  |
| e14_5_f4_source | Main source of food |  |
| e14_6_f1 | m. MILK AND MILK PRODUCTS | false |
| e14_6_f1_times_yesterday | How many of times yesterday (past 24 hours)? |  |
| e14_6_f1_source | Main source of food |  |
| e14_7_f1 | n. OILS AND FATS | false |
| e14_7_f1_times_yesterday | How many of times yesterday (past 24 hours)? |  |
| e14_7_f1_source | Main source of food |  |
| e14_7_f2 | o. RED PALM PRODUCTS | false |
| e14_7_f2_times_yesterday | How many of times yesterday (past 24 hours)? |  |
| e14_7_f2_source | Main source of food |  |
| e14_8_f1 | p. SUGAR-SWEETS | false |
| e14_8_f1_times_yesterday | How many of times yesterday (past 24 hours)? |  |
| e14_8_f1_source | Main source of food |  |
| e16_how_long_after_birth_put_to_breast | E 16. How long after birth did you/mother first put (NAME) to the breast? |  |
| e19_still_breastfeeding | E 19. Is (NAME) still breastfeeding currently? |  |
| e20_breastfeeding_stopped_at | E 20. If (NAME) is not still breastfeeding, at what age in months did you stop? |  |
| e21_times_of_breastfeeding | E 21. Last night, how many times did you breastfeed (NAME) from sunset to sunrise? |  |
| e23_anything_dink_in3days_after_delivery | E 23. In the first 3 days after delivery, was [NAME] given anything to drink other than breast milk? |  |
| e24_list_reported | E 24. If yes tick ALL items that are reported. Simply record all liquids mentioned. Do not read the list of possible responses |  |
| e26_a | A Plain water? |  |
| e26_a_times | HOW MANY TIMES/INSHURO |  |
| e26_b | B Infant formula such as [Nan, Guigoz, Nido, etc]? |  |
| e26_b_times | HOW MANY TIMES/INSHURO |  |
| e26_c | C Milk such as tinned, powdered, or fresh animal milk? |  |
| e26_c_times | HOW MANY TIMES/INSHURO |  |
| e26_d | D Juice or juice drinks? |  |
| e26_d_times | HOW MANY TIMES/INSHURO |  |
| e26_e | E Clear broth? |  |
| e26_e_times | HOW MANY TIMES/INSHURO |  |
| e26_f | F Yogurt? |  |
| e26_f_times | HOW MANY TIMES/INSHURO |  |
| e26_g | G Thin porridge? |  |
| e26_g_times | HOW MANY TIMES/INSHURO |  |
| e26_h | H Any other liquids such as [inzoga, ikigage, etc]? |  |
| e27_how_many_times_eat_other_than_liquids | E 27. How many times did [NAME] eat solid, semi-solid or soft foods other than liquids yesterday during the day or at night? (question for children >6 months) |  |
| e28_1 | a. Bread, rice, noodles/makaroni, or other foods made from grains, including thick grain-based porridge? |  |
| e28_2 | b. White potatoes, white yams/amateke ibikoro, manioc, cassava, or any other foods made from roots? |  |
| e28_3 | c. Pumpkin, squash, carrots, or sweet potatoes that are yellow or orange inside? |  |
| e28_4 | d. Any foods made from beans, peas, lentils or nuts, including Plumpy 'nut? |  |
| e28_5 | e. Any dark green leafy vegetables? |  |
| e28_6 | f. Any other fruits or vegetables? |  |
| e28_7 | g. Any red meat such as beef, pork, lamb, goat, chicken or duck? |  |
| e28_8 | h. Any white meat such as chicken or duck or other birds? |  |
| e28_9 | i. Fresh or dried fish, or seafood? |  |
| e28_10 | j. Eggs? |  |
| e28_11 | k. Cheese, yogurt, or milk, and other milk products? |  |
| e28_12 | l. Any oil, fats or butter, or foods made with any of these? |  |
| e28_13 | m. Any sugary foods such as chocolates, sweets, candies, pastries, cakes or biscuits? |  |
| e28_15 | o. Any milk/animal source food from own farm? |  |
| e16_17_any_plumpy_nut | q. Any plumpy nut? |  |
| e29_who_prepated_meals | E 29. During the last two weeks, who prepared most of the meals for the child? |  |
| e31_times_child_fed_by_others | E 31. During the last two weeks, how many days was the child fed by someone other than mother and father? |  |
| e33_given_vitamina_capsule | E 33. Was your baby given any vitamin A capsule in the last 6 months? |  |
| e34_received_deworming_tablets | E 34. Has (name of the child) received deworming tablets in the last 6months |  |
| e35_ b used_multiple_micronutrient_powder | E 35 b. Have you used multiple micronutrient powder to add to the foods of (name of the child) in the last week? |  |
| e37_received_plumpy_nut | E 37. Did the child receive plumpy nut /RUTF (Ready to Use Therapeutic Food) in the last 3 months? |  |
| e37_b | E 37b. Did the child receive fortified food (Shishakibondo) in the last 2 weeks? |  |
| e38_ever_attended_nutrition_clinic | E 38. Did your child ever attend a nutrition clinic like Supplementary feeding programme or Therapeutic feeding centre? |  |
| e40_nutrition_education_in_village | E 40. Is there any nutrition education and counselling program in the community (village)? |  |
| e41_you_attend_any_nutrition_education | E 41. In the last month, did you attend any session on nutrition education and counselling |  |
| e44_times_of_growth_monitoring | E 44. How many times did your child receive growth monitoring measurement in the last 6 months? |  |
| e45_received_nutrition_support | E 45. Have you ever received any support in nutrition program? |  |
| e46_type_of_received_services | E 46. If Yes, what type of services did you receive? |  |
| e47_hh_livestock | E 47. Does the household keep livestock? |  |
| e47_hh_livestock_type | If yes, what types of livestock do you keep? |  |
| e48_hh_have_home_garden | E 48. Does the household have a home garden |  |
| e49_type_of_vegetables_in_garden | E 49. What types of vegetables and nutrient dense crops (bio-fortified crops) are grown in the field |  |
| e49_a | E 49.a. Which of the following nutrient dense crops has your household ever grown? |  |
| e50_type_of_fruit_trees | E 50. What type of fruit trees are grown by the household? |  |
| e51_how_use_garden_products | E 51. How do you use the kitchen/home garden produces? |  |
| e52_main_source_food_household_consumption | E 52. How do you get access to the food that your household consumes? |  |
| f1_farm_size | F 1. What is your average farm size owned for agricultural activities? |  |
| f2_livestock | F 2. What types of livestock do you keep? |  |
| f3_milk_product | F 3. What type of milk product do you take at your house? |  |
| f4_make_fermented_milk | F 4. If you take fermented milk, how do you make it? |  |
| f5_milk_teatment_before_consumption | F 5. What type of (milk) treatment do you perform before consumption? |  |
| f6_boiling_duration | F 6. How long do you boil milk before drinking? |  |
| f7_keeping_milk_before_boiling_duration | F 7. How long do you keep the milk before boiling? |  |
| f8_boiled_milk_container | F 8. In which type of containers do you keep boiled milk? |  |
| g1_1_prevents_seeing_friends | a. He tries to keep you from seeing your friends |  |
| g1_3_restricts_contact_family | b. He tries to restrict contact with your family of birth |  |
| g1_5_ignores_treats_indifferntly | c. He ignores you and treats you indifferently |  |
| g1_7_gets_angry_speaking_another_man | d. He gets angry if you speak with another man |  |
| g1_9_suspicious_that_unfaithful | e. He is often suspicious that you are unfaithful |  |
| g1_11_expects_permission_for_health_care | f. He expects you to ask his permission before seeking health care for yourself |  |
| g1_13_controls_how_spend_money | g. He controls how you spend your money |  |
| g2_a_a | a. Insulted you or made you feel bad about yourself |  |
| g2_a_b | -Did this happen at any time in your life before the pregnancy period with the child NAME? |  |
| g2_a_c | -Did this happen to you during the pregnancy or after your child NAME was born? |  |
| g2_b_a | Belittled or humiliated you in front of other people? |  |
| g2_b_b | -Did this happen in the year before the pregnancy with the child NAME? |  |
| g2_b_c | -Did it happen to you during the pregnancy or after your child NAME was born? |  |
| g2_c_a | Done things to scare or intimidate you on purpose? |  |
| g2_c_b | -Did this happen in the year before the pregnancy with the child NAME? |  |
| g2_c_c | -Did this happen to you during the pregnancy or after your child NAME was born? |  |
| g2_d_a | Threatened to hurt someone you care about? |  |
| g2_d_b | -Did this happen in the year before the pregnancy with the child NAME? |  |
| g2_d_c | -Did this happen to you during the pregnancy or after your child NAME was born? |  |
| g2_more | More information to add related to above |  |
| g3_a_a | a. Slapped you or thrown something at you that could hurt you? |  |
| g3_a_b | -Did this happen at any time in your life before the pregnancy period with the child NAME? |  |
| g3_a_c | -Did this happen to you during the pregnancy or after your child NAME was born? |  |
| g3_b_a | b. Pushed you or shoved you? |  |
| g3_b_b | -Did this happen at any time in your life before the pregnancy with the child NAME? |  |
| g3_b_c | -Did this happen to you during this pregnancy period or after your child NAME was born? |  |
| g3_c_a | c. Hit you or shoved you? |  |
| g3_c_b | -Did this happen at any time in your life before the pregnancy with the child NAME? |  |
| g3_c_c | -Did this happen to you during this pregnancy period or after your child NAME was born? |  |
| g3_d_a | d. Kicked you, dragged you or beaten you up? |  |
| g3_d_b | -Did this happen at any time in your life before the pregnancy with the child NAME? |  |
| g3_d_c | -Did this happen to you during this pregnancy period or after your child NAME was born? |  |
| g3_e_a | e. Choked or burnt you on purpose? |  |
| g3_e_b | -Did this happen at any time in your life before the pregnancy with the child NAME? |  |
| g3_e_c | -Did this happen to you during this pregnancy period or after your child NAME was born? |  |
| g3_f_a | f. Threatened to use or actually used a gun, knife or other weapon against you? |  |
| g3_f_b | -Did this happen at any time in your life before the pregnancy with the child NAME? |  |
| g3_f_c | -Did this happen to you during this pregnancy period or after your child NAME was born? |  |
| g3_more | More information to add related to above |  |
| g4_a_a | a. Did your current or former husband/partner ever physically force you to have sexual inter-course when you did not want to? |  |
| g4_a_b | -Did this happen at any time in your life before the pregnancy period with the child NAME? |  |
| g4_a_c | -Did this happen during this pregnancy period or after your child NAME was born? |  |
| g4_b_a | Did you ever have sexual intercourse you did not want to because you were afraid of what your current or former husband/partner might do? |  |
| g4_b_b | -Did this happen at any time in your life before the pregnancy with the child NAME? |  |
| g4_b_c | -Did this happen during this pregnancy period or after your child NAME was born? |  |
| g4_c_a | Did your current or former husband/partner ever force you to do something sexual that you found degrading or humiliating? |  |
| g4_c_b | -Did this happen at any time in your life before the pregnancy with the child NAME? |  |
| g4_c_c | -Did this happen to you during this pregnancy period or after your child NAME was born? |  |
| g5_violence_from_others_during_pregnancy | G 5. Has any other person than your husband used physical or sexual violence towards you during your latest pregnancy? |  |
| g6_who | G 6. If 'YES' to the above question, who was this person? |  |
| g7_1_had_cuts_bruises_aches | a. You had cuts, bruises, or aches? |  |
| g8_afraid_of_husband | G 8. Are you afraid of your (last) husband/partner? |  |
| g10_1 | 1. Took away privileges, forbade something the he/she/Name liked, or did not allow him/her/Name to leave the house (child NAME). |  |
| g10_2 | *2. Did this happen to any other child/children in the family?* |  |
| g10_5 | 5. Shook him/her (NAME of the child NAME). |  |
| g10_6 | *6. Did this happen to any other child/children in the family?* |  |
| g10_7 | 7. Shouted, yelled at or screamed at him/her/Name (child NAME). |  |
| g10_8 | *8. Did this happen to any other child/children in the family?* |  |
| g10_9 | 9. When he/she/NAME did something wrong, did you or any other adult in the household give him/her/NAME something else to do? |  |
| g10_10 | *10. Did this happen to any other child/children in the family?* |  |
| g10_11 | 11. Hit him/her on the bottom with bare hand |  |
| g10_12 | *12. Did this happen to any other child/children in the family?* |  |
| g10_13 | 13. Hit him/her on the bottom or elsewhere on the body with something like a belt, hairbrush, stick or other hard object (child NAME/Name). |  |
| g10_14 | *14. Did this happen to any other child/children in the family?* |  |
| g10_15 | 15. Called him/her dumb, lazy or another name like that (child NAME/Name). |  |
| g10_16 | *16. Did this happen to any other child/children in the family?* |  |
| g10_21 | 21. Beat him/her up, that is hit him/her over and over as hard as one could (child NAME/Name). |  |
| g10_22 | *22. Did this happen to any other child/children in the family?* |  |
| g11_do_believe_in_children_physical_punishment | G 11. Do you believe that in order to bring up, raise, or educate a child properly, the child needs to be physically punished? |  |
| h1_today_overal_health | H 1. Would you say your overall health today is: |  |
| h2_6 | 6. Anxiety |  |
| h2_7 | 7. Depression |  |
| h2_8 | 8. Headache |  |
| h2_9 | 9. Fatigue, extreme tiredness |  |
| h3_1 | 1. Tuberculosis |  |
| h3_2 | 2. HIV/AIDS |  |
| h3_3 | 3. Malaria |  |
| h3_4 | 4. Low body weight, being too slim |  |
| h3_5 | 5. High blood pressure |  |
| h3_13 | 13. Diarrhoea |  |
| h3_14 | 14. Intestinal worms |  |
| h3_20_other | Any other disease, please specify: |  |
| h4_somke_before_pregnancy | H 4. Before the pregnancy with the child NAME, did you smoke tobacco? If Yes please indicate how many times do you smoke per day. |  |
| h5_smoke_during_pregnancy | H 5. Did you smoke any cigarettes during the pregnancy with the child NAME? If Yes please indicate how many times do you smoke per day |  |
| h6_alcohol_before_pregnancy | H 6. Did you use any form of alcohol before the pregnancy with the child NAME? |  |
| h7_alcohol_during_pregnancy | H 7. Did you use any form of alcohol during the pregnancy with the child NAME? |  |
| h9_how_often_husband_drinks | H 9. How often does your husband/partner drink alcohol? |  |
| h_a1 | *A1. Have you been consistently depressed or down, most of the day, nearly every day, for the past two weeks?* |  |
| h_a2 | *A2. In the past two weeks, have you been less interested in most things or less able to enjoy the things you used to enjoy most of the time?* |  |
| h_a3_a | a. Was your appetite decreased or increased nearly every day or did your weight decrease or increase without trying intentionally? |  |
| h_a3_b | b. Did you have trouble sleeping nearly every night (difficulty falling asleep, waking up in the middle of the night, early morning wakening, or sleeping excessively)? |  |
| h_a3_c | c. Did you talk or move more slowly than normal or were you fidgety, restless or having trouble sitting still, almost every day? |  |
| h_a3_d | d. Did you feel tired or without energy, almost every day? |  |
| h_a3_e | e. Did you feel worthless or guilty, almost every day? |  |
| h_a3_f | f. Did you have difficulty concentrating or making decisions, almost every day? |  |
| h_a3_g | g. Did you repeatedly consider hurting yourself, feel suicidal, or wish that you were dead? |  |
| h_a4 | A4. MAJOR DEPRESSIVE EPISODE CURRENT |  |
| h_a5_a | A5 a. During your lifetime, did you have other periods of two weeks or more when you felt depressed or uninterested in most things, and had most of the problems we just talked about? |  |
| h_a5_b | A5 b. Was there an interval of at least 2 months without depression and/or lost of interest between your current episode and your last episode of depression? |  |
| h_a5 | A5. MAJOR DEPRESSIVE EPISODE PAST |  |
| h_b1 | B1. Do you think that you would be better off dead or wish you were dead? |  |
| h_b2 | B2. Do you want to harm yourself? |  |
| h_b3 | B3. Do you think about suicide? |  |
| h_b4 | B4. Do you have a suicide plan? |  |
| h_b5 | B5. Do you attempt suicide? |  |
| h_b6 | B6. In your life time, did you ever make a suicide attempt? |  |
| h_b | SUICIDE RISK CURRENT |  |
| h_c1_a | *C1 a. Have you worried excessively or been anxious about several things of day to day life, at work, at home, in your close circle over the past 6 months?* |  |
| h_c1_b | *C1 b. Are these worries present most days?* |  |
| h_c2 | C2. Do you find it difficult to control the worries or do they interfere with your ability to focus on what you are doing? |  |
| h_c3_a | a. Feel restless, keyed up or on edge? |  |
| h_c3_b | b. Feel tense? |  |
| h_c3_c | c. Feel tired, weak or exhausted easily? |  |
| h_c3_d | d. Have difficulty concentrating or find your mind going blank? |  |
| h_c3_e | e. Feel irritable? |  |
| h_c3_f | f. Have difficulty sleeping (difficulty falling asleep, waking up in the middle of the night, early morning wakening or sleeping excessively)? |  |
| h_c3 | GENERALIZED ANXIETY DISORDER CURRENT |  |
| i1_decision_major_purchases | I 1. Who usually makes decisions about making major household purchases? |  |
| i2_decision_visit_family | I 2. Who usually makes decisions about visits to your family, relatives and friends? |  |
| i3_decision_on_earned_money | I 3. Who usually decides how the money you earn will be used? |  |
| i4_own_house | I 4. Do you own this or any other house either alone or jointly with someone else? |  |
| i5_own_any_land | I 5. Do you own any land either alone or jointly with someone else? |  |
| i6_who_make_decision_abou_health | I 6. Who usually makes decisions about health care for yourself? |  |
| i7_influences_on_family_planning | I 7. Who or what influences your decision on family planning the most? |  |
| i8_can_say_no_to_sexual_intercourse | I 8. Can you say no to your husband/partner if you do not want to have sexual intercourse? |  |
| i9_can_ask_use_candom | I 9. Could you ask your (husband/partner) to use a condom if you wanted him to? |  |
| j_01 | Capture a photograph of the "immunisation card document", "house", or other important documents. |  |
| j_02 | If you need, you could record a short interview (less than five minutes). |  |
| k1_land_ownership | K 1. Type of land ownership |  |
| k3_watering_livestock | K 3. Is water for watering livestock available in the area? |  |
| k4_water_sources | K 4. Sources of water available in the area |  |
| k5 | K 5. Frequency of offering water to animals |  |
| k6 | K 6. If “No”, why? |  |
| k7_month_water_livestock | K 7. Which months do you experience water shortage for watering your livestock (including fodder production)? |  |
| k8_annual_water_quality | K 8. What is water quality used in your farm? |  |
| k9_water_quality_test | K 9. How do you test the quality of water? |  |
| k10_livestock _feed_shortage | K 10. Do you experience feed shortage for your livestock? |  |
| k11 | K 11. If “Yes” why? |  |
| k12_month_feed_shortage | K 12. Indicate which months do you experience feed shortage? |  |
| k13 | K 13. Availability of mineral block |  |
| k14 | K 14. If “Yes”, where did you get it |  |
| k15 | K 15. If “No”, why? |  |
| k16_purchase_feed | K 16. Do you purchase feed? |  |
| k17_feed_purchase_types | K 17. If yes, indicates the major type of feed purchased per year. |  |
| k18_annually_feed_purchased | K 18. Indicate the cost of feed purchased annually? |  |
| k19_planted_forages_fodder | K 19. Do you have planted forages/fodder? |  |
| k20_farm_crops | K 20. If Yes, indicate types of crops grown in your farm |  |
| k21_forage_decision_maker | K 21. Who makes decision on the type of forage to plant/grow? |  |
| k22_forage_place_in_farm | K 22. Where in your farm do you plant your forages? (several response alternatives possible) |  |
| k23_forage_area_dictator | K 23. What dictates the area you plant fodder/forages? |  |
| k24_fertilizer_forages | K 24. Do you apply fertilizer to your forages |  |
| k25_fertilizer | K 25. If yes which one? |  |
| k26_f18_why_not | K 26. If no why not? |  |
| k27_feed_conserve_type | K 27. How do you conserve feed for your livestock? |  |
| k28 | K 28. Who are involved in grass hay, silage production? |  |
| k29_why_not_forages | K 29. Why don't you have planted forages? |  |
| k30_crop_residues_used_for | K 30. Uses of crop residues from the farm |  |
| k31 | K 31. If fed to livestock, how do you treat the crop residues for quality improvement? |  |
| k32 | K 32. If you use urea, what are the impact in livestock production? |  |
| k33_collect_fodder_for_livestock | K 33. Do you collect or gather green fodder to feed your livestock |  |
| k34_other_fodder_benefits | K 34. If “NO”, list other benefit(s) of green fodder. |  |
| k35_processed_feed_for_livestock | K 35. Do you use homemade concentrates for livestock in your farm? |  |
| k36_f26_what_feed | K 36. If “YES”, what feed do they use? |  |
| k37_f26_why_no | K 37. If No, why no? |  |
| k38_keeping_record_at_farm | K 38. Do you keep record at your farm? |  |
| k39_what_records | K 39. If 'YES' what records do you keep? |  |
| k40_farming_system | K 40. What farming system are you practicing? |  |
| k41_reproduction_technique | K 41. What type of reproduction technique do you use in your farm? |  |
| k42_get_mating_bull_from | K 42. If natural mating. Where do you get the bull? |  |
| k35_have_land_for_forage | K 35. In case of extensive system. Do you have enough land for forage production? |  |
| k43_animal_house_floor | K 43. Type of animal house floor |  |
| k44_feeding_style | K 44. What is the style of feeding? |  |
| k45 | K 45. If open grazing, where do you graze your animal? |  |
| k46_animal_house_cleaning_period | K 46. How often do you clean the animal house? |  |
| k47_milking_cows_number | K 47. How many milking cows do you have? |  |
| k48_milking_times_per_day | K 48. How many times per day do you milk? |  |
| k49_milking_technique | K 49. What milking technique do you uses? |  |
| k50_produced_ltrs_per_farm_per_day | K 50. How many liters do you produce per farm per day? |  |
| k51_lactation_period | K 51. For how long do your cows produce milk per lactation? |  |
| k52_how_proceed_milking | K 52. How do you proceed when milking? |  |
| k53_milking_containers | K 53. Which type of milking containers do you use? |  |
| k54_where_do_milk | K 54. Where do you milk from? |  |
| k55_do_screen_subclinical_mastitis | K 55. Do you screen for subclinical mastitis? |  |
| k56_how_do_ensure_milk_quality | K 56. How do you ensure milk quality in your herd? (several response alternatives possible) |  |
| k57_milk_production_purpose | K 57. What is the main purpose of your milk production? |  |
| k58_where_do_sell_milk_product | K 58. Where do you sell your milk produce? |  |
| k59_treatment_before_consumption | K 59. What type of (milk) treatment do you perform before consumption? |  |
| k60_howlong_boil_milk | K 60. How long do you boil milk before drinking? |  |
| k61_when_do_wash_containers | K 61. When do you wash milking containers? |  |
| k62_water_source_for_cleaning_utensils | K 62. From where do you get water for cleaning milking utensils? |  |
| k63_water_type_for_cleaning_equipment | K 63. What type of water do you use to clean milking equipment? |  |
| k64_howlong_to_deliver_milk | K 64. How long does it take you to deliver milk to your client(s)? |  |
| k65_container_type_for_transportation | K 65. Which type of containers do you use for milk transportation? |  |
| k66_means_of_transport | K 66. What means of transport do you use to deliver your milk produce? |  |
| k67_experienced_milk_rejection | K 67. Did you ever experience milk rejection? |  |
| k68_rejection_reasons | K 68. If yes, what were reasons for milk rejection? |  |
| k69_what_do_with_rejected_milk | K 69. What do you do with the rejected milk? |  |
| k70_diseases_experienced_in_herds | K 70. What are the animal diseases do you experience in your herd(s)? |  |
| k71_reproductive_diseases_in_herds | K 71. What reproductive diseases do you experience in your herd? |  |
| k72_do_know_animal_human_disease_transmit | K 72. Do you know that animals may transmit diseases to humans? |  |
| k73_how_think_transmit | K 73. If yes, how do you think this transmission may occur? |  |
| k74_which_disease_think_can_transmit_human | K 74. Which animal disease do you think can be transmitted to humans? (Several response alternatives possible) |  |
| k75_how_prevent_these_disease | K 75. How can these disease be prevented in humans? (Several response alternatives possible) |  |
| k76 | K 76. Have any household member had any gastrointestinal symptoms during the last 2 weeks? |  |
| k77 | K 77. If “yes” what kind of symptoms? |  |
| k78 | K 78. Do you know what caused these symptoms? |  |
| k79 | K 79. If “yes” what are the causes? |  |
| k80_what_prevention_mechanisms_know | K 80. What are the mechanisms of disease prevention in animals do you know? (Several response alternatives possible) |  |
| k81_how_newanimals_introduced_herd | K 81. How are new animals introduced in the herd? |  |
| k81_1_quarantine_days | If quarantine, specify the duration in days |  |
| k81_2_what_do_to_animals_in_quarantine | If quarantine, what do you do to the animals during that time? |  |
| k82_where_get_veterinary_services | K 82. Where do you get veterinary services from (when your animals are sick)? |  |
| k83_what_do_chronic_sick_animals | K 83. What do you do when you have a chronic sick animal? |  |
| k84_what_do_with_milk_from_sickanimals | K 84. What do you do with the milk from sick animals (under treatment)? |  |
| k85_previous_animal_keeping_experience | K 85. Did you or any household member have previous experience in animal keeping? |  |
| k86_had_training_farming_production_management | K 86. Have you had any training on farming production management? |  |
| k87_what_module_trained | K 87. If yes, what module(s) have you been trained on? |  |
| k88_improvement_done_after_trainings | K 88. What improvement did you make after these trainings? |  |

END
