# Supplementary material for: Association between poor mental health in mothers and child stunting: a population-based cross-sectional study in Rwanda
Source: BMJ Open. 2025 Oct 13;15(10):e101117. doi: 10.1136/bmjopen-2025-101117 (PMC12519718; doi:10.1136/bmjopen-2025-101117)
Supplement: online supplemental table 1 [file bmjopen-15-10-s003.docx]

**Supplemental Table 1** Sociodemographic characteristics of the overall study population and categorized by mental health disorders (*N*=601)

| **Mothers’ characteristics** | **All respondents, *n* (%)** | **Mothers with different types of mental health disorders, *n* (%)** | | | | | | | |
| --- | --- | --- | --- | --- | --- | --- | --- | --- | --- |
|  |  | **Current major depression disorders, *n* (%)** | ***p* value** | **Recurrent major depression disorders, *n* (%)** | ***p* value** | **Current suicide risk, *n* (%)** | ***p* value** | **Current generalized anxiety disorder, *n* (%)** | ***p* value** |
| Number (%) | 601 | 135 (22.7) |  | 81 (27.3) |  | 108 (18.2) |  | 138 (36.6) |  |
| **Mother’s age** |  |  |  |  |  |  |  |  |  |
| ≤20 years | 30 (5.0) | 11 (8.1) | 0.153 | 7 (8.6) | 0.719 | 7 (6.5) | 0.701 | 13 (9.4) | 0.024 |
| 21–34 years | 352 (58.6) | 74 (54.8) |  | 45 (55.6) |  | 64 (59.3) |  | 75 (54.4) |  |
| ≥35 years | 219 (36.4) | 50 (37.0) |  | 29 (35.8) |  | 37 (34.3) |  | 56 (36.2) |  |
| **Education** |  |  |  |  |  |  |  |  |  |
| Never attended school | 58 (9.7) | 17 (12.6) | 0.446 | 7 (8.6) | 0.138 | 7 (6.5) | 0.370 | 11 (8.0) | 0.417 |
| Primary level | 412 (68.5) | 90 (66.7) |  | 51 (63.0) |  | 74 (68.5) |  | 95 (68.8) |  |
| Secondary level and above | 131 (21.8) | 28 (20.7) |  | 23 (28.4) |  | 27 (25.0) |  | 32 (23.2) |  |
| **Marital status** |  |  |  |  |  |  |  |  |  |
| Married | 531 (88.3) | 116 (85.9) | 0.344 | 65 (80.3) | 0.052 | 90 (83.3) | 0.083 | 120 (87.0) | 0.590 |
| Single, widowed, or divorced | 70 (11.7) | 19 (14.1) |  | 16 (19.7) |  | 18 (16.7) |  | 18 (13.0) |  |
| **Occupation** |  |  |  |  |  |  |  |  |  |
| Skilled | 11 (1.9) | 1 (0.8) | 0.463 | 0 (0.0) | 0.450 | 3 (2.9) | 0.415 | 2 (1.5) | 0.274 |
| Student | 6 (1.0) | 2 (1.5) |  | 2 (2.6) |  | 2 (1.9) |  | 4 (3.0) |  |
| Non-skilled | 574 (97.1) | 128 (97.7) |  | 76 (97.4) |  | 98 (95.2) |  | 128 (95.5) |  |
| **Household income** |  |  |  |  |  |  |  |  |  |
| ≥36,000 RWF**^a^** | 95 (16.0) | 12 (9.0) | 0.014 | 5 (6.3) | 0.002 | 9 (8.5) | 0.022 | 12 (8.8) | 0.003 |
| <36,000 RWF | 500 (84.0) | 122 (91.0) |  | 75 (93.7) |  | 97 (91.5) |  | 125 (91.2) |  |
| **Child’s age** |  |  |  |  |  |  |  |  |  |
| 1–12 months | 215 (35.8) | 31 (31.1) | 0.177 | 26 (32.1) | 0.736 | 41 (38.0) | 0.864 | 55 (39.9) | 0.215 |
| 13–24 months | 213 (35.4) | 46 (34.1) |  | 31 (38.3) |  | 36 (33.3) |  | 45 (32.6) |  |
| 25–36 months | 173 (28.8) | 47 (34.8) |  | 24 (29.6) |  | 31 (28.7) |  | 38 (27.5) |  |
| Child’s sex |  |  |  |  |  |  |  |  |  |
| Female | 311 (51.7) | 69 (51.1) | 0.828 | 38 (46.9) | 0.492 | 58 (53.7) | 0.685 | 69 (50.0) | 0.907 |
| Male | 290 (48.3) | 66 (48.9) |  | 43 (53.1) |  | 50 (46.3) |  | 69 (50.0) |  |
| **Child’s birth weight** |  |  |  |  |  |  |  |  |  |
| ≥2.5 kg | 533 (89.7) | 114 (86.4) | 0.163 | 69 (86.2) | 0.425 | 90 (84.9) | 0.081 | 117 (85.4) | 0.401 |
| <2.5 kg | 61 (10.3) | 18 (13.6) |  | 11 (13.8) |  | 16 (15.1) |  | 20 (14.6) |  |
| **Child breastfeeding duration according to age** |  |  |  |  |  |  |  |  |  |
| ≤12 months | 197 (98.5) | 37 (18.8) | 0.496 | 22 (11.2) | 0.581 | 36 (18.3) | 0.501 | 47 (23.8) | 0.417 |
| 13–24 months | 197 (92.9) | 43 (21.8) | 0.775 | 31 (15.7) | 0.259 | 39 (19.8) | 0.101 | 46 (23.3) | 0.950 |
| 25–36 months | 57 (30.2) | 16 (28.1) | 0.895 | 8 (14.0) | 0.898 | 9 (15.8) | 0.643 | 9 (15.8) | 0.118 |

**^a^**RWF, Rwandan Francs.
