# Supplementary material for: Association between poor mental health in mothers and child stunting: a population-based cross-sectional study in Rwanda
Source: BMJ Open. 2025 Oct 13;15(10):e101117. doi: 10.1136/bmjopen-2025-101117 (PMC12519718; doi:10.1136/bmjopen-2025-101117)
Supplement: Supplementary file 4 [file bmjopen-2025-101117.reviewer_comments.pdf]

PEER REVIEW HISTORY

BMJ Open publishes all reviews undertaken for accepted manuscripts. Reviewers are asked to complete a checklist review form and are provided with free text boxes to elaborate on their assessment. These free text comments are reproduced below.

ARTICLE DETAILS

Title (Provisional)

Association between poor mental health in mothers and child stunting: a population-based cross-sectional study in Rwanda

Authors

Utumatwishima, Jean Nepo; Mogren, Ingrid; Elfving, Kristina; Umubyeyi, Aline; Krantz, Gunilla

---

VERSION 1 - REVIEW

**Reviewer**	**1**
**Name**	**Khan, Ahad**
**Affiliation**	**The University of Edinburgh, Usher Institute**
**Date**	**28-May-2025**
**COI**	**None**

General Comments:

This is a well-conducted study examining the link between maternal mental health and child stunting in a resource-limited setting. The study addresses an important public health issue and is methodologically sound, with a large sample size and use of validated tools. However, few revisions are required to enhance the clarity and completeness of the manuscript, especially regarding the abstract and methodological details. These are outlined below.

Abstract Section:

Please revise the abstract to follow the BMJ Open structured format, including the subheadings: Objective, Design, Setting, Participants, Primary and Secondary Outcome Measures, Results and Conclusions.

Methods Section:

Line 122–123: Please consider moving this sentence to the beginning of a new paragraph to improve logical flow and readability.

Line 159: Please elaborate on HAZ at first mention.

Line 179: Please specify how the child's birth weight was collected. Was it self-reported by the mother, or retrieved from health cards or medical records?

Line 233: Please detail how the HAZ values were used to create the binary stunting variable.

Missing Data: Please clarify whether any data were missing for key variables. If so, provide information on how missing data were handled (e.g., listwise deletion, imputation).

**Reviewer**	**2**
**Name**	**Khaliq, Asif**
**Affiliation**	**Queensland University of Technology**
**Date**	**16-Jul-2025**
**COI**	**None**

---

1) Add (LMICs) after low-and-middle income countries

2) The word Mental health is a broad term, can you please narrow down ?

3) Use word stunting rather than nutritional status.

4) Write full form of DSM, WHO, HAZ, and SD in the abstract

5) The word high is not an appropriate word. In the scientific writing, our focus is on the facts and figures.

6) You need to show that among four different types of mental disorder, you found highest prevalence of GAD followed by . . . .

7) Abstract results -This all seems repetitive . .. Summarize it and present in more meaningful fashion

8) I think you did a cross sectional study, so I think it would be prudent to measure the odds rather than relative risk, because the RR better fits for the longitudinal studies.

9) Rather than saying high showed a significant association or a relationship of maternal mental health and stunting was observed.

10) You need to write proper definition of stunting.

11) Mother's characteristics, including education, weight, height, and antenatal care attendance, along with factors like sanitation, family size, birth order, birth weight, breastfeeding practices, cooking environment, and socioeconomic status, are linked to child stunting . . . . How these factors are linked to stunting? Are they protective or causative? I suggest you to describe the direction. Which practice, behaviour or factor can cause stunting or which can protect stunting. . .. You need to research this more

12) An Istanbul-based study reveals an association between poor maternal mental health and an increased risk of emotional neglect and/or physically violent behaviours towards young children . . . . This sentence is not relevant to your study.

13) The rational of study is very weak, because you already presented there is a relation of stunting or nutritional adversities with poor maternal mental health, then why there is need to study?

14) Content of all the studies presented in the introduction - German, Peru, Rio de Janeiro and Rwanda should be part of discussion.

15) You need to show why assessing the relationship of maternal mental health with stunting is important and why we need to work on these measure?

16) Poor maternal mental health and stunting both are the problems but you did not explain either of these in an appropriate manner in your introduction.

17) What is the rational to choose children aged between 1 to 36 months?

Ideally, stunting is a chronic form of malnutrition that takes around 3 months to develop . . .

Why you did not consider children below 5 years?

Alternatively you can do work only on children aged below 2 years . . .

18) If CI is 95% then margin of error should be 5%.

19) Response rate should be part of result: The participation rate was 95.4%. There were incomplete data for 29 mother–child pairs.

20) Write the reference of your questionnaire otherwise attach your questionnaire in the appendix or supplementary file.

21) I did not understand in the abstract you wrote you used DSM for assessing maternal health but in the manuscript methdology this seems different???

22) Why you measured the weight? Child weight has no relationship with the paediatric stunting.

23) Remove the content related to wasting and underweight.

24) I did not understand why child weight was used as a covairate. Remove it. ~ If you are considering birthweight then how all the mothers remember their child birthweight? Rwanda is a LMIC and I am not sure the about the recall and about birth weight measurement in all the healthcare setting. How many domicially deliveries? these factors are not addressed anywhere in the methodolgy and discussion. You needs to discuss this.

25) full form of IPV, ICD, MINI . . . etc

26) Write all the maternal mental health condition methods in separate headings - also attach the questionnaire in the appendix

27) Content of the ethical consideration needs to be summarise

28) Total is 601, and 72 individual = 33.2% how ??

29) Remove table 5

30) Citations are missing in the dicussion.

31) The discussion part is extremely weak. You need to discuss in the light of previous literature.

32) Need to add more limitations of the study.

33) Conclusion is overly stated.

**** The reviewer provided a marked copy with additional comments. Please contact the publisher for full details.****

VERSION 1 - AUTHOR RESPONSE

Reviewer 1

Thank you for your comments.

For abstract: *Thank you for your observation, I have fixed the abstract following BMJ Open structured format.*

Methods section

Line 122-123: *This line talks about....**A total number of 630 households**...We have move it. The information about the participation rate was moved to results according to the second reviewer.*

Line 159: Yes, Height-for-age Z scores (HAZ). It was fixed in the new revised document.

Line 179: *Thank you. In the revised document, I have added the following sentence: "The child's birth weight, **recorded in kilograms on the vaccination card**, was categorized as low birth weight (<2.5 kg) and normal birth weight (≥2.5 kg)". All 601 children had vaccination cards. Vaccination in Rwanda is mandatory, every child has a vaccination card and it is reinforced by community health workers.*

Line 233: Thank you for this addition. The following line was added:

"*Height-for-age Z scores (HAZ) were used to create a binary stunting variable. Children with HAZ values below −2 standard deviations were classified as stunted (stunting = 1), while those with HAZ ≥ −2 were classified as not stunted (stunting = 0). Missing HAZ values were handled using listwise deletion, meaning that any observation with missing data on HAZ or relevant variables was excluded from analyses involving the stunting variable". (in Stata we used this code: gen stunting =.*
replace stunting = 1 if haz < -2
replace stunting = 0 if haz >= -2).

Reviewer 2

1) **Yes, fixed. …..***Child undernutrition is linked to substantial national economic and health losses in low- and middle-income countries (LMICs), ….*

2) **The word: mental health:** *This study aims to determine the prevalence of major depressive disorders, generalized anxiety, and suicide risk among mothers in Rwanda and to explore their association with child stunting.*

3) **Use word stunting:** *Yes fixed.*

4) **DSM, WHO, HAZ, and SD: Yes fixed. ….***Mothers' mental health was assessed using four modules from the Mini International Neuropsychiatric Interview (MINI), based on the Diagnostic and Statistical Manual of Mental Disorders, Fourth Edition (DSM-IV), version 5.0.0. Child anthropometric measurements followed World Health Organization (WHO) guidelines.*

5) **The word high:** Yes improved in the abstract

6) **You need to show that among four different types of mental disorder, you found highest prevalence of GAD followed by . . . .**: *This has been fixed in the abstract.*

7) **Abstract results -This all seems repetitive . .. Summarize it and present in more meaningful fashion:** *Yes, this has been fixed in the abstract.*

8) I think you did a cross sectional study, so I think it would be prudent to measure the odds rather than relative risk, because the RR better fits for the longitudinal studies:

Thank you for this observation. *Although this was a cross-sectional study where odds ratios are standard, we also calculated Excess Relative Risk (ERR) to complement our analysis and provide a clearer picture of the relative contribution of mothers' mental health conditions to child stunting. ERR was computed by comparing risk levels across models: a baseline model (including sociodemographic variables) and successive models adding generalized anxiety disorder, current major depression, suicide risk, and their combinations. This allowed us to estimate the additional risk attributable to each condition beyond the baseline. While ERR does not imply causality in a cross-sectional design, it serves as a useful descriptive measure to highlight the relative burden of mothers' mental health on child nutritional outcomes.*

9) Rather than saying high showed a significant association or a relationship of maternal mental health and stunting was observed: *This was fixed.*

10) You need to write proper definition of stunting: Thank you, I have written it like this: *Child stunting, also known as chronic undernutrition, is defined as a height-for-age Z-score below −2 standard deviations from the median of the WHO child growth standards and affects 22% of all children in the world.*

11) Mother's characteristics, including education, weight, height, and antenatal care attendance, along with factors like sanitation, family size, birth order, birth weight, breastfeeding practices, cooking environment, and socioeconomic status, are linked to child stunting . . . . How these factors are linked to stunting? Are they protective

or causative? I suggest you to describe the direction. Which practice, behaviour or factor can cause stunting or which can protect stunting. . .. You need to research this more.

Yes, Thank you for this observation. I have shown the direction in the text as follows*: ----Mothers' characteristics such as low education, low weight and height, and inadequate antenatal care attendance are risk factors that increase the likelihood of child stunting. Similarly, poor sanitation, large family size, higher birth order, low birth weight, suboptimal breastfeeding practices, unsafe cooking environments, and low socioeconomic status contribute to a higher risk of stunting.*

12) An Istanbul-based study reveals an association between poor maternal mental health and an increased risk of emotional neglect and/or physically violent behaviours towards young children . . . . This sentence is not relevant to your study.

"Thank you for your insightful comment. We agree that the sentence referencing the Istanbul-based study on emotional neglect and physically violent behaviors is not directly aligned with the primary focus of our study, which centers on maternal mental health and child stunting. To maintain clarity and focus in the introduction, we have removed this sentence in the revised manuscript."

13) The rational of study is very weak, because you already presented there is a relation of stunting or nutritional adversities with poor maternal mental health, then why there is need to study?

Thank you for your helpful comment. We agree that it is important to clarify the rationale for studying the link between mothers' mental health and child stunting.

While international studies have established this association, our introduction explains that no previous research in Rwanda has assessed this relationship using population-based data.
At the same time, depression and anxiety are highly prevalent among mothers in Rwanda (e.g., 37.6% and 28.2% in one study), and stunting remains a major public health issue, with a prevalence of 34.1% in children aged 6–23 months from poor households. Moreover, current national guidelines do not include mothers' mental health as part of stunting prevention or management, despite evidence that mental health affects caregiving, nutrition, and health-seeking behaviors.
This represents a major gap in both research and policy. Our study addresses this gap and aims to generate context-specific evidence to inform integrated, evidence-based interventions that could support Rwanda's efforts to reduce stunting to 19% by 2024.
We have revised the introduction to make this rationale clearer, as suggested.

14) Content of all the studies presented in the introduction - German, Peru, Rio de Janeiro and Rwanda should be part of discussion. *"Thank you for this suggestion, we have referred to it in the discussion"*

15) You need to show why assessing the relationship of maternal mental health with stunting is important and why we need to work on these measure? *We have revised the introduction to make this rationale clearer, as suggested.*

16) Poor maternal mental health and stunting both are the problems but you did not explain either of these in an appropriate manner in your introduction: *We have revised the introduction to make this rationale clearer, as suggested.*

17) What is the rational to choose children aged between 1 to 36 months?
Ideally, stunting is a chronic form of malnutrition that takes around 3 months to develop . . .
Why you did not consider children below 5 years?
Alternatively you can do work only on children aged below 2 years . . .

Thank you for your valuable comment. We selected the age range of 1–36 months to capture both early and ongoing risk of stunting during a biologically vulnerable period. While the first 1,000 days (up to 24 months) are critical, evidence shows that growth faltering can continue beyond 24 months, especially in low-resource settings.
We excluded children under 1 month to avoid confounding from birth-related factors, and those over 36 months to reduce bias from external influences (e.g., daycare, community programs, dietary diversity) and ensure closer alignment between mothers' current mental health and child growth outcomes.
We have clarified this rationale in the manuscript.

18) If CI is 95% then margin of error should be 5%.

Thank you for your valuable feedback regarding the margin of error. While a 5% margin of error is commonly used and generally accepted in prevalence studies, it is not an absolute requirement. The choice of margin of error depends on the study's objectives, desired precision, and available resources. For our study, we selected a 4% margin of error to enhance the precision of our estimates while maintaining a feasible sample size within our resource constraints. We believe this choice appropriately balances the need for accuracy with practical considerations and is consistent with accepted scientific standards.

We appreciate the opportunity to clarify this point.

19) Response rate should be part of result: The participation rate was 95.4%. There were incomplete data for 29 mother–child pairs: "*Thank you for your helpful suggestion. We agree that the participation rate and information on incomplete data are better suited for the Results section, as they describe the actual data collected and sample characteristics. We will move this sentence accordingly in the revised manuscript".*

20) Write the reference of your questionnaire otherwise attach your questionnaire in the appendix or supplementary file: ***Yes, I will add it in supplementary file.***

21) I did not understand in the abstract you wrote you used DSM for assessing maternal health but in the manuscript methdology this seems different*???: **Yes, MINI questionnaire is built based on the DSM criteria.***

22) Why you measured the weight? Child weight has no relationship with the paediatric stunting: ***Yes, very clear. We removed weight.***

23) Remove the content related to wasting and underweight: ***Yes removed.***

24) I did not understand why child weight was used as a covairate. Remove it. ~ If you are considering birthweight then how all the mothers remember their child birthweight? Rwanda is a LMIC and I am not sure the about the recall and about birth weight measurement in all the healthcare setting. How many domicially deliveries? these factors are not addressed anywhere in the methodolgy and discussion. You needs to discuss this.

Thank you for your insightful comment regarding the inclusion of birthweight as a covariate. While stunting is defined by length/height-for-age, birthweight remains an important early-life factor that influences later growth trajectories and nutritional status.
Low birthweight reflects intrauterine growth restriction or prematurity, both of which predispose children to growth faltering and stunting during infancy and early childhood. Adjusting for birthweight helps control for these early biological risks, allowing us to better isolate the association between mothers' mental health and subsequent child stunting. Therefore, including birthweight as a covariate strengthens the validity of our analysis by

In our study, birth weight data were obtained from the child's vaccination card for all 601 participants. In Rwanda, every child is systematically enrolled in the national immunization program, and vaccination cards are routinely provided and retained by families. This is made possible through the country's strong vaccination system and the active involvement of community health workers, who ensure follow-up even for children born at home. This approach supports the reliability of birth weight data across all delivery settings.

25) full form of IPV, ICD, MINI . . . etc: Yes, fixed.

26) Write all the maternal mental health condition methods in separate headings - also attach the questionnaire in the appendix: Yes, fixed.

27) Content of the ethical consideration needs to be summarise: Yes, summarized.

28) Total is 601, and 72 individual = 33.2% how ??

Thank you very much for your thoughtful question regarding the percentage reported for the group of 72 (33.2%). We appreciate the opportunity to provide further clarification.

The percentage originates from the Venn diagram, which illustrates the overlapping and distinct distributions of the three mental health disorders assessed in our study: depression, anxiety, and suicide risk. The total number of mothers who screened positive for at least one of these conditions is ***n = 217****.*

This total represents the sum of all mutually exclusive categories, as follows:

- *Depression only: 33 (15.2%)*
- *Anxiety only: 4 (1.8%)*
- *Suicide risk only: 38 (17.5%)*
- *Depression + Anxiety (without suicide risk): 72 (33.2%)*
- *Anxiety + Suicide risk (without depression): 40 (18.4%)*
- *Depression + Suicide risk (without anxiety): 8 (3.7%)*
- *All three disorders: 22 (10.1%)*

Thus, the total number of mothers with any of these conditions is:
33 + 4 + 38 + 72 + 40 + 8 + 22 = 217.

Accordingly, the percentage for the group with Depression + Anxiety only is calculated as:
(72 ÷ 217) × 100 = 33.2%.

We hope this explanation addresses your query, and we remain grateful for your careful review.

29) Remove table 5:

Thank you for your valuable feedback regarding the presentation of the data in Table 5. In response to your suggestion, we have removed the table as advised. However, we have retained the key findings in the text to ensure that the association between maternal depression symptoms and child nutritional status remains clearly communicated to readers.

We appreciate your careful review and guidance, which have helped us improve the clarity and conciseness of the manuscript.

30) Citations are missing in the discussion: Yes, fixed
31) The discussion part is extremely weak. You need to discuss in the light of previous literature: Yes, fixed according to your suggestions from the introduction.

32) Need to add more limitations of the study: ***Yes, I added the following:*** *. The exclusion of mothers with missing anthropometric or mental health data through listwise deletion may have led to selection bias, potentially affecting the generalizability of the findings. The study was also limited to rural settings, which may not reflect the situation in urban areas, where risk factors and health service access could differ.*

33) Conclusion is overly stated.

Thank you for your observation. The conclusion is summarized and concise.

We thank you again for the opportunity to revise our work. We are grateful for the reviewers' time and insightful comments, which have helped us significantly improve the quality and clarity of our manuscript.
